# The regulatory code of injury-responsive enhancers enables precision cell-state targeting in the CNS

**DOI:** 10.1038/s41593-025-02131-w

**Published:** 2025-12-02

**Authors:** Margherita Zamboni, Adrián Martínez-Martín, Gabriel Rydholm, Timm Häneke, Laura Pintado Almeida, Deniz Seçilmiş, Christoph Ziegenhain, Enric Llorens-Bobadilla

**Affiliations:** 1 Department of Cell and Molecular Biology, Karolinska Institute, Stockholm, Sweden; 2 SciLifeLab, Department of Gene Technology, KTH Royal Institute of Technology, Stockholm, Sweden; 3 Department of Medical Biochemistry and Biophysics, Karolinska Institute, Stockholm, Sweden

**Keywords:** Astrocyte, Molecular neuroscience, Spinal cord injury, Epigenetics and plasticity

## Abstract

Enhancer elements direct cell-type-specific gene expression programs. After injury, cells change their transcriptional state to adapt to stress and initiate repair. Here we investigate how injury-induced transcriptional programs are encoded within enhancers in the mammalian CNS. Leveraging single-nucleus transcriptomics and chromatin accessibility profiling, we identify thousands of injury-induced, cell-type-specific enhancers in the mouse spinal cord after a contusion injury. These are abundant in glial cells and retain cell-type specificity, even when regulating shared wound response genes. By modeling glial injury-responsive enhancers using deep learning, we reveal that their architecture encodes cell-type specificity by integrating generic stimulus response elements with cell identity programs. Finally, through in vivo enhancer screening, we demonstrate that injury-responsive enhancers can selectively target reactive astrocytes across the CNS using therapeutically relevant gene delivery vectors. Our decoding of the principles of injury-responsive enhancers enables the design of sequences that can be programmed to target disease-associated cell states.

## Abstract (web summary)

Zamboni et al. reveal how enhancers encode cell-type-specific responses to CNS injury. By combining multiomic profiling, deep learning and in vivo screening, they uncover injury-responsive enhancer logic and enable targeting of reactive astrocytes.

## Main

Enhancer regulatory elements in the genome control precise patterns of gene expression essential for tissue development and homeostasis. Enhancer sequences contain transcription factor binding sites that encode the information to unfold cell-type-specific gene expression programs^1^. These regulatory sequences can direct expression of their target genes even outside their natural genomic context^2–4^. These properties have made enhancers uniquely useful experimentally to control cell-type-specific expression in gene delivery systems and reporter assays. Indeed, large efforts have been devoted to identifying cell-type-specific elements to develop experimental tools for specific cell targeting and gene therapy applications^5–9^. However, no systematic effort has been made to define injury-responsive enhancers (IRENs) and their sequence properties in the mammalian CNS.

The CNS contains the highest degree of cell-type specialization, encompassing many neurons and glial subtypes. This specialization, in turn, leads to a heterogeneous response to injury, particularly in glial cells, and recent studies have begun to characterize injury- or disease-associated cell states and the transcriptional programs characterizing them^10–12^. How the spatiotemporal regulation of these gene expression programs across different cell types is encoded within genomic regulatory elements remains, however, largely elusive.

Leveraging single-nucleus multiomic approaches, we sought to unravel the regulatory logic that governs the activation of injury-dependent transcriptional programs in the injured mouse spinal cord. We found that injury responses were prominent in glial cells. Both unique and shared injury-induced programs in glia were associated with newly commissioned sets of cell-type-specific IREN elements. By training sequence-to-function deep learning models across cell types and states, we decoded the syntax of IRENs, revealing that these DNA elements encode cell-state specificity by directing simultaneous recruitment of generic stimulus-responsive and lineage-specific transcription factors, an architecture we validate in vivo. Finally, we used our models’ predictive power to identify regulatory elements that could drive gene expression selectively in reactive astrocytes at CNS damage sites using systemic viral vectors.

## Results

### A multimodal cell census of the injured spinal cord

**Fig. 1:**
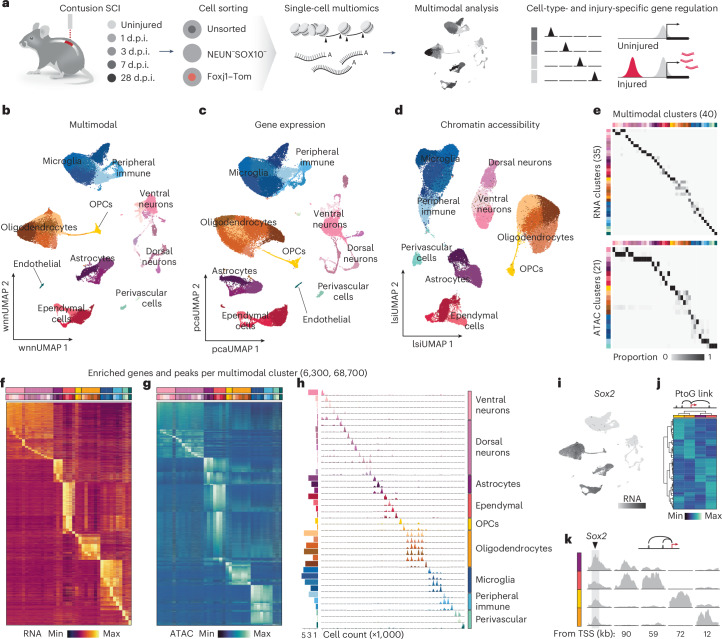
A glia-enriched multimodal cell census of the injured spinal cord. **a**, Schematic workflow of the multiomic experiment. Mice were subjected to a contusion injury to the spinal cord, and tissue was collected at different time points after the injury, as well as from uninjured controls. We isolated nuclei from whole tissue (unsorted) as well as from samples enriched in less abundant cell populations using flow cytometry: neuron and oligodendroglia depleted (NEUN^−^SOX10^−^) or ependymal cell enriched (Foxj1–tdTomato (Foxj1–Tom)). The nuclei were then profiled at the multiomic level (ATAC + RNA) to investigate injury-responsive gene regulatory programs; SCI, spinal cord injury. **b**–**d**, Uniform manifold approximation and projection (UMAP) visualization and clustering calculated on multiomic (**b**), gene expression (**c**) and chromatin accessibility data (**d**); wnn, weighted nearest neighbor; pca, principal component analysis; lsi, latent semantic indexing. **e**, Heat map displaying clustering agreement, in proportion, between analyses computed on multiomic and gene expression data (top), and multiomic and chromatin accessibility data (bottom). The number of populations identified at the same clustering resolution is reported in parentheses for each modality. **f**,**g**, Heat map displaying average scaled data for significantly enriched genes (**f**) and peaks (**g**) for each cluster identified at the multiomic level. Colored bars represent cell types (top) and subtypes (bottom). **h**, Coverage plots from examples of enriched peaks across the multiomic clusters. On the left, bar plots display cluster size (×1,000), whereas on the right, tracks are grouped according to cell type. **i**, Feature plot displaying gene expression levels for *Sox2*. **j**, Heat map representing average scaled accessibility across glia cells for peaks linked to *Sox2* (peak-to-gene (PtoG) links). **k**, Coverage tracks for the *Sox2* transcription start site (TSS), along with distal regions enriched in different glial cells. Distance from the TSS (in kilobases (kb)) is reported below the plots.

**Extended Data Fig. 1:**
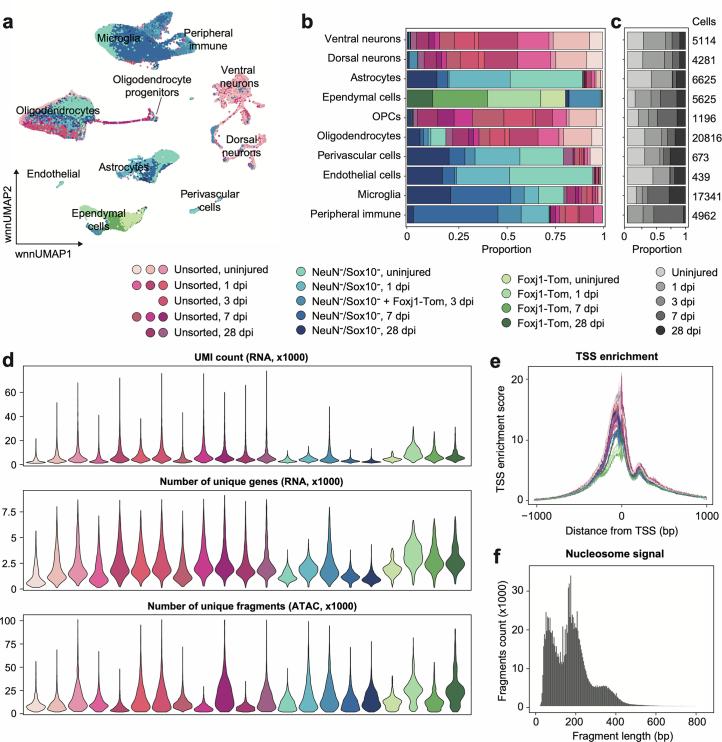
Overview of the multiomic dataset and quality metrics. **a**, UMAP visualization of the multiome dataset colored by sample. Nuclei originate from one of three sample types: all nuclei (unsorted), depleted of neurons and oligodendrocytes (NEUN and SOX10 depleted), or enriched in ependymal cells (Foxj1-Tom sorted), as well as a timepoint after injury. **b**, Cell type abundance color-coded according to nuclei sample origin and timepoint. **c**, Cell type abundance across timepoints. Includes nuclei from all samples. **d**, Violin plots displaying number of UMI counts, number of genes and number of fragments for the transcriptome and chromatin accessibility data, respectively. Plots are colored by sample of origin. **e**, Mean enrichment score around the transcription start site (TSS) for each sample. **f**, Fragment size histogram.

To study the regulatory programs underlying the transcriptional response to spinal cord injury, we conducted a simultaneous single-nucleus transcriptomics and chromatin accessibility profiling experiment (RNA sequencing (RNA-seq) and assay for transposase-accessible chromatin (ATAC) using sequencing (ATAC-seq); Fig. 1a and Extended Data Fig. 1). For this purpose, we isolated nuclei from the lesion site of mice subjected to mid-thoracic contusion injury and sampled them at relevant time points to resolve temporal dynamics during acute (1 and 3 days postinjury (d.p.i.)), subacute (7 d.p.i.) and chronic (28 d.p.i.) phases (Fig. 1a).

Given the abundance of oligodendrocytes and neurons in the spinal cord^10^, whose response to injury has been broadly investigated in previous transcriptional studies^11,13^, we implemented enrichment strategies to steer the collection of injury-responsive cell types that are less comprehensively characterized (Extended Data Fig. 1a,b). To do this, in addition to isolating nuclei from the whole tissue to define the unbiased cell census, we isolated nuclei using flow cytometry from samples depleted of the most abundant cell types (that is, based on NEUN and SOX10 expression) and from a transgenic reporter mouse line to enrich for rare ependymal cells^14,15^. This allowed us to increase the sampling rate of non-neuronal cells, including astrocytes, ependymal cells and immune cells (Extended Data Fig. 1a,b). Cumulatively, our curated dataset consisted of 67,072 high-quality nuclei and encompassed all major resident neuronal and non-neuronal cell types, as well as local and infiltrating immune cells (Fig. 1b–d). Each cell type was represented across experimental conditions, except for peripheral immune cells, which predominantly populate the tissue in the acute and subacute phases^16,17^ (Extended Data Fig. 1a–c). The cell census and population sizes of this dataset agree with previously published work^11,13^.

Using nearest neighbor-based clustering, each major lineage could be computationally segregated into fine-grained subtypes and states (Fig. 1b–e), characterized by specific sets of enriched features (Fig. 1f–h). At the same clustering resolution, our multimodal data identified a larger number of subpopulations (40 clusters) than either modality alone (35 and 21 clusters for RNA-seq and ATAC-seq, respectively; Fig. 1e). Importantly, even at this higher level of granularity, subtypes and states were unequivocally defined by sets of enriched genes (6,300 unique genes at a *P* value of <0.05 and log_2_ (fold change) (log (FC)) of >0.5; Fig. 1f), as well as a tenfold larger number of differentially accessible regions (68,700 unique regions at a *P* value of <0.05 and log_2_ (FC) of >0.5; Fig. 1g,h and Supplementary Fig. 1). Over 80% of differentially accessible regions corresponded to distal *cis*-regulatory elements, with fewer mapping to less variable promoter regions (Supplementary Fig. 1b). These *cis*-regulatory elements are largely composed of putative enhancers, and, despite their genomic identification, for simplicity and given their later validation, we will hereafter refer to these elements as ‘enhancers’ (Supplementary Fig. 1c).

Reflecting the rich regulatory landscape, our differential expression analysis could also reveal examples of marker genes shared across several cell types, such as *Sox2* (for example, all macroglia types), but whose expression was associated with unique sets of enhancers in each lineage (Fig. 1i–k and Supplementary Fig. 2a). Similarly, cell-type-specific enhancer accessibility could discern cell types with highly shared marker gene expression, such as pericytes and oligodendrocyte progenitor cells (OPCs; Supplementary Fig. 2b–d).

Thus, our dataset combining chromatin accessibility with gene expression in single nuclei offers a rich resource to investigate not only the molecular identity of the cell types populating the mouse spinal cord but also the repertoire of lineage-specific regulatory elements.

### Deep learning models predict cell-type-specific regulatory syntax

**Fig. 2:**
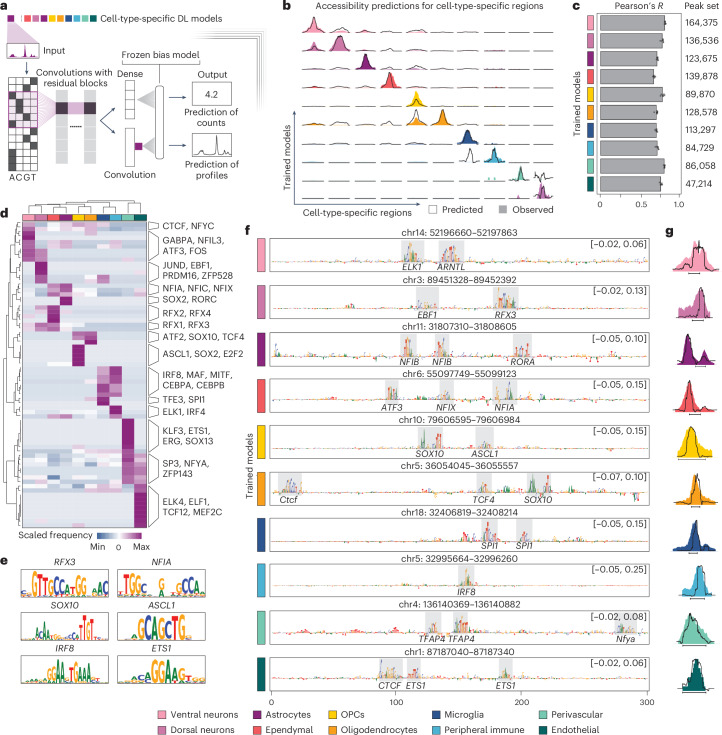
Deep learning models predict accessibility and regulatory logic across cell types. **a**, Schematic overview of the architecture of the deep learning (DL) models used to predict chromatin accessibility across cell types from the whole dataset. The models are trained for each cell type using cell-type-specific peak sets and process their one-hot encoded DNA sequences through a series of dilated convolutions with residual connections. The final output generates count predictions and profile log-likelihood via a dense layer or an additional convolutional layer, respectively. **b**, Coverage plots displaying observed (colored area) and predicted (black line) accessibility for cell-type-enriched genomic regions. Each peak is predicted using a different cell-type-specific model (rows). **c**, Bar plots reporting Pearson’s correlations between observed and predicted counts for each cell-type-specific model. Error bars report standard deviations calculated from the correlations obtained with fivefold cross-validations. On the right, the number of peaks used as input for each model is reported. **d**, Heat map highlighting the frequency (scaled by row) of top occurring motifs in sets of peaks called for each cell type. On the right, examples of transcription factors recognizing such sequences are reported. **e**, Sequence logos for representative DNA binding sites and their matching transcription factors. **f**, Nucleotide contribution scores calculated by each cell-type-specific model for representative genomic regions. Motifs identified carrying higher importance are highlighted (gray), and the transcription factors recognizing such sequences are reported under the colored area. The limits on the *y* axis are reported in brackets. **g**, Observed (colored area) and predicted (black line) accessibility profiles of the regions reported in **f**.

Thanks to the dense sampling of cell populations and genome-wide profiling of their chromatin accessibility landscape, our dataset provided us with a unique resource to infer the transcription factor motif syntax of lineage-specific regulatory elements in the spinal cord using deep learning^18–22^. Interpretable convolutional neural networks, such as ChromBPNet, are particularly suited to decode the regulatory logic underlying enhancer accessibility, given their ability to recognize salient features in DNA sequences de novo (that is, transcription factor binding sites) and their higher-order architecture^18,19,22,23^. We, thus, trained models for each major cell type to learn their accessibility profiles based on the DNA sequence at base-pair resolution (Fig. 2a). We then leveraged the models’ interpretability to identify transcription factor motifs associated with cell-type-specific regulatory element accessibility^22^. The models achieved high predictive power for accessibility tracks of held-out sequences (Fig. 2b and Supplementary Fig. 3a) and good correlation between observed and predicted counts across the genome in all cell types (Pearson’s *R* ranging between 0.675 ± 0.013 for ependymal cells and 0.820 ± 0.007 for ventral neurons; Fig. 2c).

**Extended Data Fig. 2:**
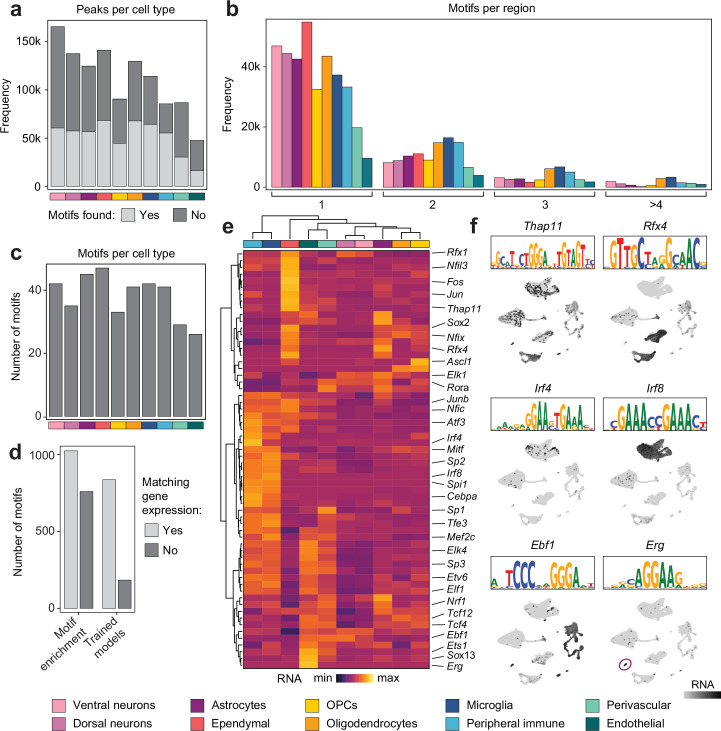
Analysis of motif enrichment across cell types. **a**, Number of peaks used for training the cell type specific models from the complete dataset. Bars are colored based on whether regions carry motifs identified by the models. **b**, Number of cell type-specific regions carrying one or more motifs detected by the trained models. **c**, Number of unique motifs identified by the trained models in each set of cell type-specific peaks. **d**, Total number of motifs identified using traditional motif enrichment analysis compared to our trained models. Bars are split based on whether motifs match expression of the transcription factor in the correct cell type. **e**, Heatmap displaying the average scaled gene expression levels of transcription factors for which our trained models found enriched motifs in the cell type specific peaks (linked to Fig. 2d). **f**, Feature plots displaying gene expression of representative transcription factors, along with the sequence logos corresponding to their DNA binding sites.

We, therefore, leveraged the models’ ability to deduct important features from regulatory DNA sequences to nominate motifs enriched across different cell types (Fig. 2d and Extended Data Fig. 2). Using DeepLift^24^ followed by TF-MoDISco^25^, we computed contribution scores for each base pair across accessible regulatory elements and inferred transcription factor binding sites from the resulting enriched motif patterns. We identified motifs in more than half of the elements examined (52 ± 5.8%; Extended Data Fig. 2a), with 32 ± 11% of regions carrying more than one motif and 3 ± 2% exhibiting more than four motifs (Extended Data Fig. 2b).

We found that our models accurately recognized lineage identity transcription factor binding sites (Fig. 2d–f and Extended Data Fig. 2c–f) and correctly predicted accessibility of elements carrying these lineage-specific motifs (Fig. 2g). For instance, genomic regions accessible in neurons were enriched in sequence motifs recognized by EBF1, whereas glial peaks were enriched in binding sites for RORC and RFX4, expressed in astrocytes and ependymal cells, and for SOX10, specific to the oligodendroglial lineage (Fig. 2d–f and Extended Data Fig. 2e). Immune cells express the transcription factor SPI1 and members of the IRF family (for example, IRF8 and IRF4), whose motifs were accurately recognized by the models (Fig. 2d–f). Finally, regulatory regions in perivascular cells featured binding sites for transcription factors, such as ERG and SOX13 (Fig. 2d), which are specifically expressed in these cell types (Extended Data Fig. 2e,f). The models also recognized that the motifs linked to ubiquitously expressed factors, such as the insulator CTCF^26^, were, in general, less variable across cell types (Fig. 2d,f). Overall, we identified 38 ± 7 consensus motif patterns per cell type, which were linked to 230 distinct transcription factors (Extended Data Fig. 2c). Cumulatively, there were 1,073 motif–transcription factor matches across cell types, 82% of them showing the corresponding cell-type-specific expression of the transcription factor (Extended Data Fig. 2d–f). By contrast, conventional motif enrichment analysis, which finds over-represented sequences in differentially accessible regions irrespective of their predicted importance, yielded only 57% of motifs with matched cell-type-specific expression of the transcription factor (Extended Data Fig. 2d).

Together, our deep learning models accurately infer chromatin accessibility from DNA sequence at base pair resolution and identify the motif syntax underlying cell-type-specific gene regulatory programs.

### Identification of cell-type-specific IRENs

**Fig. 3:**
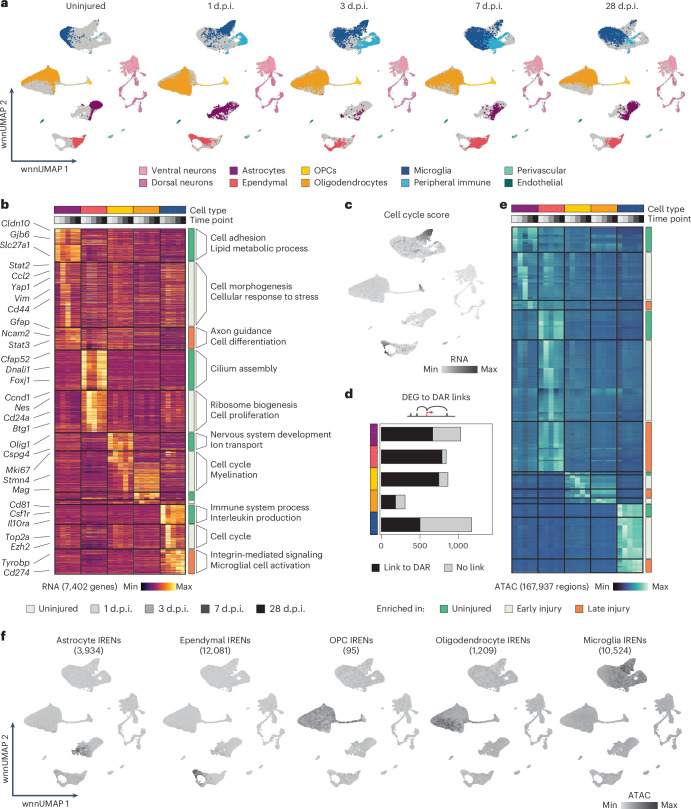
Identification of glial cell-type-specific IRENs. **a**, UMAP displaying cell types across the dataset and split by time point after spinal cord injury. **b**, Heat map reporting differentially expressed genes across time points for each of the glial cell populations. Colored bars represent cell type (top) and time point (bottom). Genes are ordered according to (1) cell-type enrichment and (2) expression dynamics and are grouped into modules depending on whether they are expressed in uninjured samples or during acute or chronic phases after injury. Left, examples of enriched genes are highlighted. Right, top enriched Gene Ontology (GO) terms are reported. Reported under the heat map is the total number of differentially expressed genes. **c**, Feature plot displaying cell cycle scores calculated for all cells across the dataset. **d**, Bar plot representing the number of differentially expressed genes (DEGs) enriched after injury across cell types. The bars are colored based on whether the genes are linked to a differentially accessible region (DAR). **e**, Heat map displaying differentially accessible peaks across cell types and time points. The grouping of regions was performed as in **b**. Reported under the heat map is the total number of differentially accessible regions recorded. **f**, Feature plots displaying combined chromatin accessibility scores of IRENs for each glial cell type. The total number of IRENs per cell type is reported in parentheses.

**Extended Data Fig. 3:**
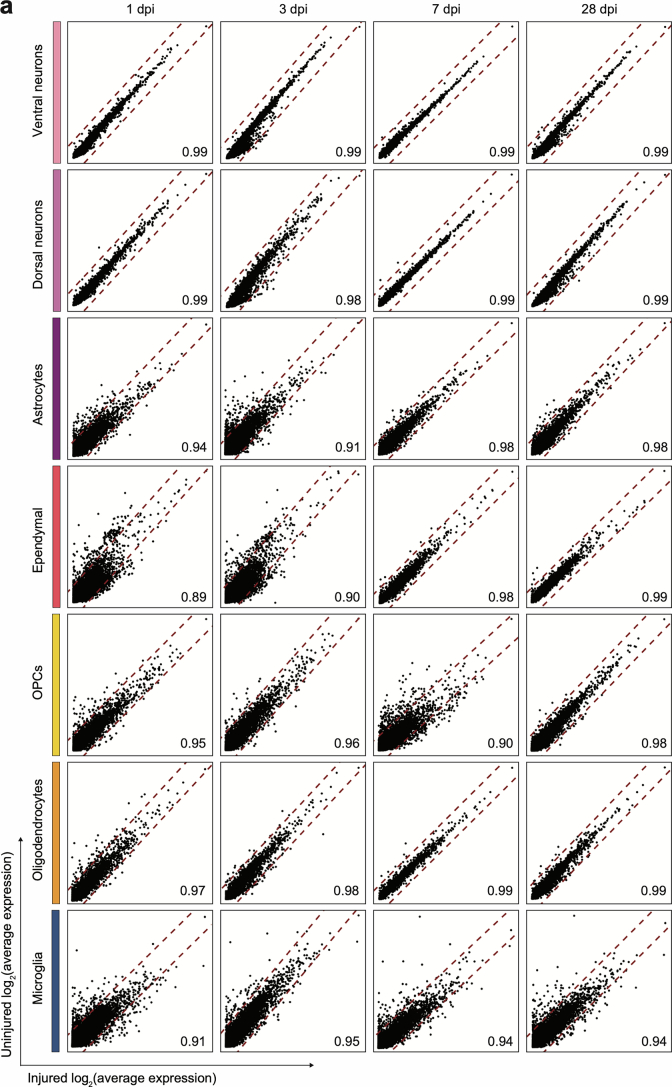
Overview of gene expression changes across time points in the glial cells. **a**, Correlation plots displaying log-transformed average expression of all genes in the dataset comparing uninjured (y-axis) with injured (x-axis) samples. Each plot represents the comparison of different timepoints (columns) and cell types (rows). Within each plot, Pearson’s correlation is reported. Dashed lines indicate a fold change > 2.

We next investigated the regulatory programs underlying injury-induced gene expression across all major spinal cord cell types (Fig. 3a). Glial cells (astrocytes, ependymal cells, OPCs and microglia) underwent more substantial transcriptional reprogramming than neurons, which remained relatively unaffected (Fig. 3a and Extended Data Fig. 3a). This prompted us to focus on glial cells.

Differential expression and accessibility analyses revealed the most pronounced alterations occurring during the early phase (1–3 d.p.i.; Fig. 3b,e and Supplementary Fig. 4a), consistent with previous observations^10^. In astrocytes, genes related to immune interactions and barrier formation (*Ccl2*, *Vim* and *Gfap*) were upregulated, and reactive astrocyte markers such as *Gfap* and *Stat3* remained elevated in later phases, indicating chronic reactivity (Fig. 3b). Peak-to-gene linkage analysis showed that most injury-induced genes were associated with differentially accessible enhancers (665 of 1,027), suggesting that astrocyte reactivity is largely driven by the commissioning of IRENs (Fig. 3d). Ependymal cells exhibited a similar pattern, with downregulation of homeostatic cilia genes (*Dnali1*) and activation of progenitor, cell cycle and migratory programs (*Nes*, *Vim* and *Cd24a*). These changes peaked at 3 d.p.i. and then mostly returned to baseline (Fig. 3b and Supplementary Fig. 4a). Most injury-induced gene expression changes (785 of 840 genes) were linked to 1,739 IRENs, with some early-commissioned enhancers remaining accessible at later stages, indicating potential epigenetic memory (Fig. 3e).

OPCs and oligodendrocytes showed a milder transcriptional response, with cell cycle-related genes activated in OPCs and myelin-related genes (*Mog*, *Mag* and *Plp1*) upregulated in oligodendrocytes (Fig. 3b,c). This was in line with previous observations recording injury-induced increases in the committed oligodendrocyte population at 7 d.p.i. (ref. ^10^). Most transcriptional changes in OPCs (744 of 862) and oligodendrocytes (184 of 308) were linked to newly accessible enhancers (Fig. 3d). Microglia showed a large transcriptional response and remained persistently reactive, but less than half of their gene expression changes (501 of 1,169) were linked to IRENs, suggesting a stronger reliance on primed regulatory elements^27^ (Fig. 3d). Importantly, even shared glial injury response genes were linked to the commissioning of cell-type-specific IRENs, suggesting a unique genome regulation of the shared modules of wound response programs (Supplementary Figs. 4b–e and 5a–e).

To characterize cell-type specificity of IRENs more broadly, we performed iterative differential accessibility analyses, retaining only regulatory elements that were injury induced in one cell type, but not in any other comparison. Combined, we obtained 27,843 injury-responsive regulatory elements that gained accessibility exclusively in the respective injury-associated cell state (Fig. 3f).

In summary, our findings reveal that glial cells reprogram their gene expression and chromatin accessibility in response to injury, a process that is largely driven by the commissioning of thousands of highly cell-type-specific IRENs.

### Decoding the regulatory syntax of IRENs using deep learning

**Fig. 4:**
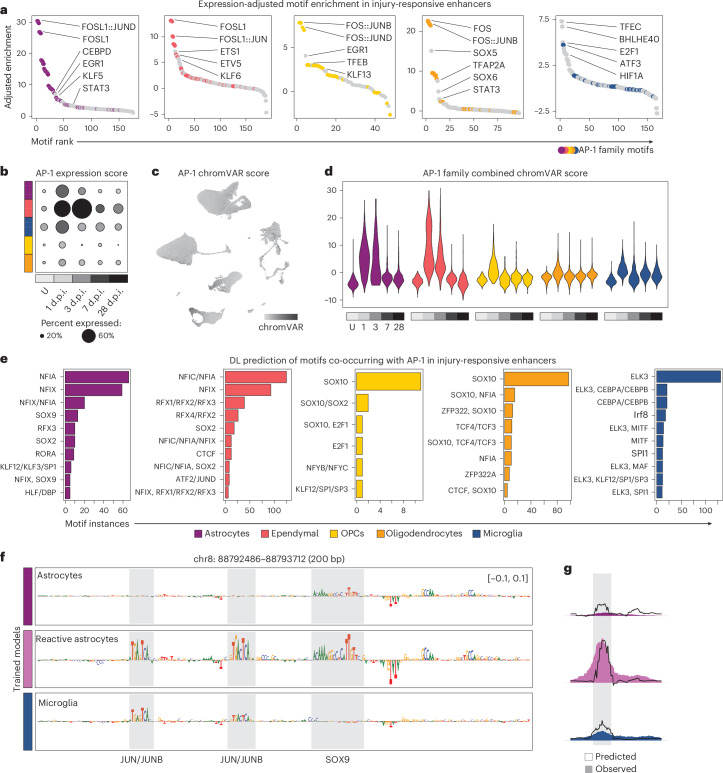
Architecture of cell-type-specific IRENs. **a**, Rank plot displaying enriched motifs in the cell-type-specific IRENs. Motif enrichment scores are adjusted by the transcription factor expression fold enrichment in the injured cell types. **b**, Dot plot reporting the module score calculated on AP-1 factors from Extended Data Fig. 4 for each glial cell type and across time points. Dots are colored based on the average score, and percentage of cells scoring above 0 is represented by the relative dot size. **c**, Feature plot displaying the combined chromVAR activity score across AP-1 factors from Extended Data Fig. 4. **d**, Violin plots showing the temporal dynamics in the combined chromVAR activity across glia cells. **e**, Bar plots reporting the number of instances each motif is co-occurring with motifs recognized by the AP-1 transcription factor family. Motif hits are based on the cell-type-specific models trained on injured samples. **f**, Nucleotide contribution scores attributed to the same astrocyte-specific, injury-responsive region. Each prediction is performed by a model trained on different sets of cells (that is, astrocytes from uninjured and injured samples and microglia). **g**, Observed (colored area) and predicted (black line) accessibility of the region in **f**. The same models from **f** are used to make predictions on the region’s accessibility across cell populations. U, uninjured.

The identification of IRENs linked to the regulation of unique and shared injury-induced gene expression programs across glial cells prompted us to study their sequence composition. Motif enrichment analysis performed on IRENs and adjusted according to the expression of the motif-linked transcription factor after injury showed a prominent enrichment of AP-1 family binding sites across glial populations (Fig. 4a). Enriched motifs with lower scores included STAT3 and CEBPD in astrocytes, which have previously been linked to the reactive astrocyte response^28,29^, EGR1 and KLF6 in ependymal cells and TFEB in OPCs, a factor previously linked to oligodendrocyte differentiation and myelination^30^. By contrast, microglia IRENs were less reliant on AP-1 factors and showed higher enrichment in motifs for a unique set of injury-induced factors, including TFEC, BHLHE40 and HIF1a (Fig. 4a). Differences in transcriptional regulatory concentration were reflected in the Gini coefficients of enriched motifs, which showed that although top-ranking AP-1 motifs accounted for the majority of differential regulatory activity in astrocytes, ependymal cells and oligodendrocytes, microglia IRENs were controlled by a more distributed network of transcription factors (Gini 0.698, 0.600 and 0.759 versus 0.585, respectively).

**Extended Data Fig. 4:**
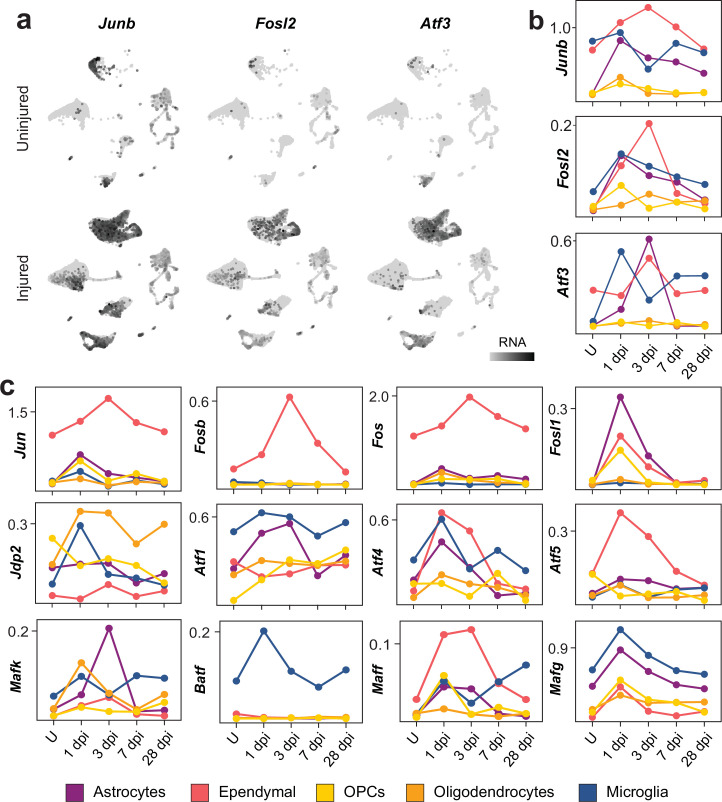
AP-1 family transcriptional dynamics in injury-responsive cells. **a**, Feature plots displaying gene expression levels of three components of the AP-1 complex. UMAPs are split by condition (that is, uninjured and injured). **b**, Average gene expression level at each timepoint after spinal cord injury for the genes reported in h. **c**, Average gene expression level at each timepoint after spinal cord injury for other genes belonging to the AP-1 transcription factor family. Only genes for which gene expression changes in an injury-dependent manner are displayed. U, uninjured.

Given the predominant enrichment of AP-1 motifs in IRENs, we explored the gene expression of AP-1 family transcription factors after injury. AP-1 factors were upregulated in all glial cells with different magnitudes of activation of specific factors (Fig. 4b and Extended Data Fig. 4a–c). These included early response genes *Junb*, *Fosl2* and *Atf3*, which were particularly prominent in astrocytes and ependymal cells, and correlated with the timing and magnitude of their global transcriptional response (Figs. 3a and 4b and Extended Data Fig. 4a–c). Consistent with their upregulation, ChromVar predicted an increased AP-1 motif usage, particularly early after injury (Fig. 4c,d). These results indicate a predominant role for AP-1 transcription factors in commissioning cell-type-specific IRENs, albeit less pronounced in microglia.

**Extended Data Fig. 5:**
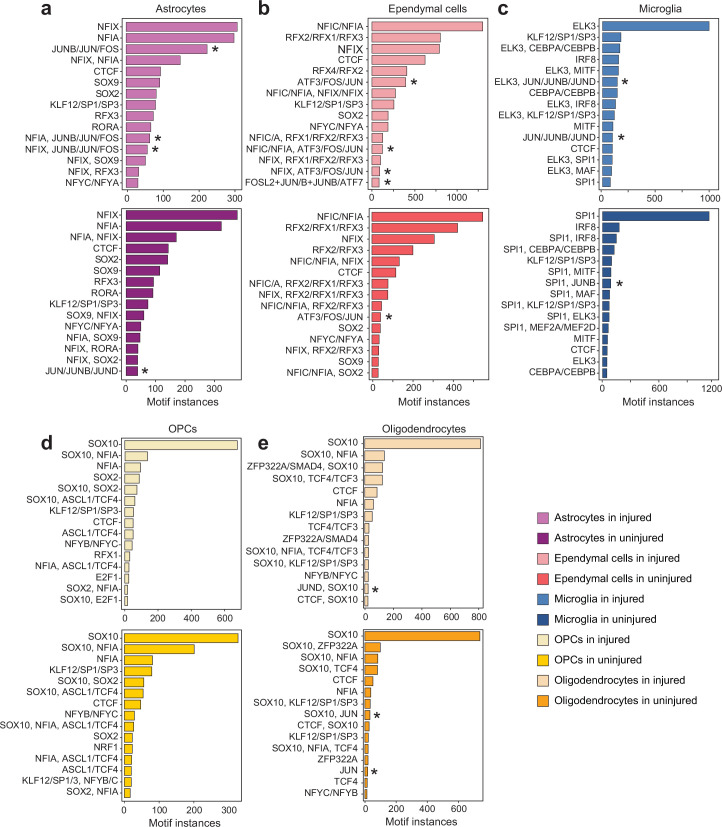
Deep learning-guided detection of motifs in cell state-specific regulatory regions. **a**–**e**, Barplot reporting the most common transcription factors and number of their motif instances recorded by the deep learning models in peaks from each glial cell type and injury condition. AP-1 transcription factors are highlighted in each barplot (*). On the y-axis, multiple transcription factors matching the same motifs are separated by slash (/), while co-occurrence of distinct motifs recognized by different genes is reported and separated by comma (,).

**Extended Data Fig. 6:**
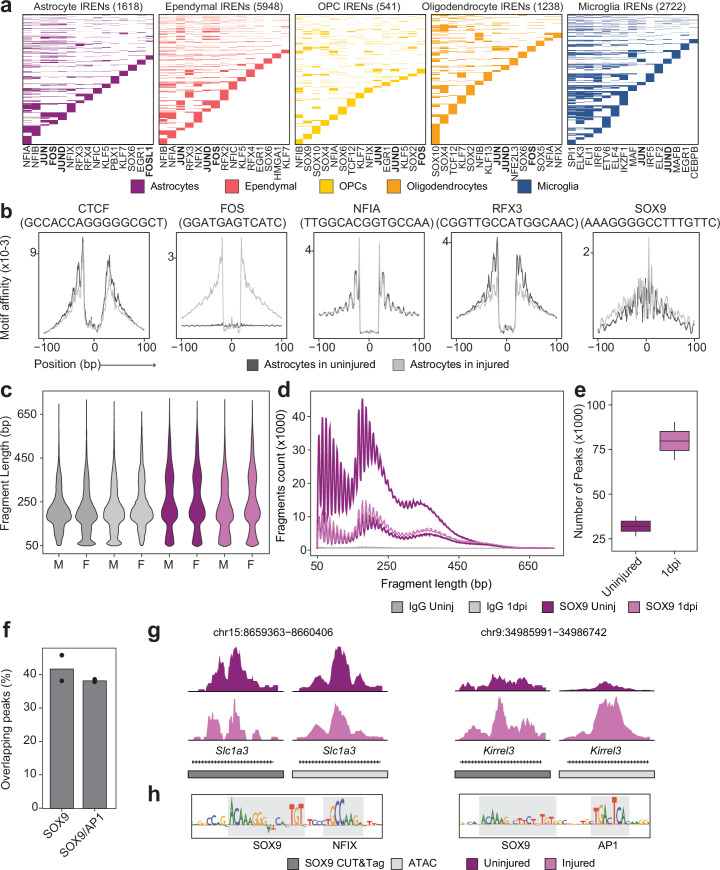
Comparison of SOX9 CUT&Tag and multiome data. **a**, Visualization of the intersection between cell type IRENs and regions belonging to enhancer regulatory networks as predicted by SCENIC+. IRENs are represented in rows, and the predicted upstream TF regulators in columns, with AP-1 factors highlighted. Region intersection indicates predicted co-regulation. **b**, Footprints predicted by the deep learning models (that is, Astrocytes in uninjured and in injured samples) for common (CTCF), injury-induced (FOS), and lineage-specific (NFIA, RFX3, SOX9) transcription factors. Curves are colored based on the trained model used to predict transcription factor binding. **c**, Violin plots reporting the fragment length distribution for each sample. Violins are colored based on the condition. Each condition was run in replicates (that is, M, male, and F, female). **d**, Plot representing the fragment size distribution for each sample. Lines are colored based on condition. **e**, Boxplots reporting the number of peaks called in each injury condition. **f**, Barplots reporting the percentage of peaks detected in the CUT&Tag experiments that overlap with regions from the multiome dataset carrying either SOX9 alone or SOX9 together with AP1 transcription factor binding motifs as predicted by the model. **g**, Coverage plots displaying accessibility in regions that are shared between uninjured and injured samples (left) or enriched in injured samples (right). Tracks are colored based on the injury condition and the color bars reported underneath represent the source of data (that is, CUT&Tag or ATAC multiome data). **h**, Nucleotide contribution scores highlighting lineage-specific only or SOX9 and AP1 motifs identified by the models in the genomic sequence of the SOX9-bound regions in **f**.

We, thus, wondered how a common set of injury-responsive transcription factors binding to the same consensus motif could lead to activating unique enhancers in different cells. Therefore, we investigated the regulatory syntax of injury-induced elements using deep learning. We trained models on injured and uninjured cell states to compare changes in the predicted contributions of de novo-identified motif representations in an unbiased manner (Extended Data Fig. 5a–e). Consistent with the results from our supervised motif enrichment analysis, most glial injury cell-state models found the consensus AP-1 binding site (TGA(G/C)TCA) among the top differentially active motifs (Extended Data Fig. 5a–e and Supplementary Table 4). To directly address how IREN selection is encoded in glia cells, we interrogated the models’ predictions to find motifs near AP-1 binding sites. We found that regulatory elements harboring AP-1 binding sites with high contribution scores most commonly also encoded motifs recognized by lineage-specific transcription factors (Fig. 4e). Lineage-specific motifs included NFI and SOX9 for astrocytes, NFI and RFX for ependymal cells, SOX10 and TCF4 for oligodendrocytes and ELK3 and CEBPA for microglia (Fig. 4e). We also confirmed the co-occurrence of lineage-specific transcription factors and AP-1 at IRENs using enhancer-based gene regulatory network analyses (Extended Data Fig. 6a)^31^. This suggests that AP-1 and lineage-specific factors cooperate to select distinct sets of IRENs in each cell type.

To further characterize the IREN architecture, we focused on IRENs specifically active in reactive astrocytes, a therapeutically relevant cell state common to multiple CNS pathologies^32,33^. The reactive astrocyte model correctly predicted state-specific IREN accessibility and identified JUN/JUNB and SOX9 binding sites as salient features, in agreement with their predicted footprints (Fig. 4f,g and Extended Data Fig. 6b). By contrast, models trained on nonreactive astrocytes or on a different reactive cell type identified motifs linked to either lineage-specific factors or stimulus-induced factors, respectively, but not in combination (Fig. 4f). Thus, our deep learning models suggest that astrocyte-specific transcription factors, such as SOX9, recruit AP-1 factors to IRENs to drive the reactive astrocyte program. To gain direct evidence of the binding of lineage-specific factors at reactive astrocyte IRENs, we performed SOX9 CUT&Tag (Extended Data Fig. 6c,d). In addition to an increase in SOX9 binding sites after injury, we observed SOX9 binding not only in ~40% of the cell identity regions predicted by the model but also in a similar percentage of the regions predicted to harbor active SOX9 and AP-1 sites (Extended Data Fig. 6e–h).

In summary, we found that the cell-type specificity of IRENs in glial cells is encoded in the DNA by directing the cooperative binding of broadly expressed, stimulus-induced AP-1 factors alongside lineage-specific transcription factors.

### Cell state targeting using IREN AAVs

**Fig. 5:**
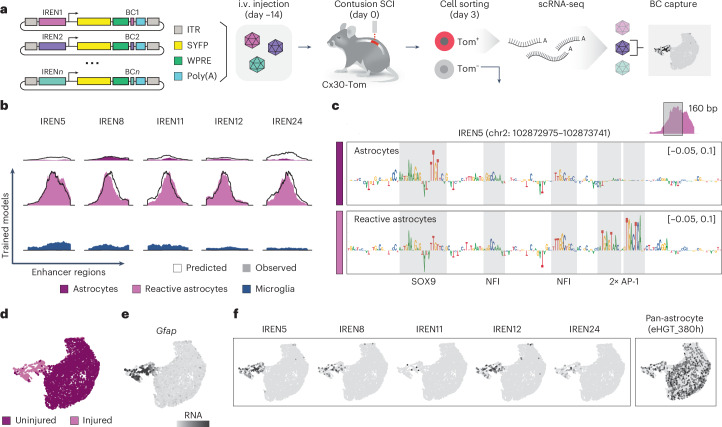
In vivo enhancer AAV screening identifies reactive astrocyte-targeting IRENs. **a**, Schematic workflow of the AAV-based reporter assay. Selected IRENs are cloned into an AAV expression cassette along with a minimal promoter^7^, a reporter transgene (SYFP), a unique barcode (BC) for AAV library demultiplexing and a poly(A) tail to enable capture on a 10x Genomics platform. CX30–tdTomato (CX30–Tom) mice were treated with tamoxifen and injected intravenously (i.v.) with the AAV library 14 days before surgery. Mice were then injured, and at 3 d.p.i. astrocytes were sorted based on tdTomato expression. Their transcriptome was profiled, and barcodes were detected computationally; scRNA-seq, single-cell RNA-sequencing. **b**, Observed (colored area) and predicted (black line) accessibility of selected enhancer regions from our screen. Each profile is predicted by different cell-type- and state-specific models (rows). **c**, Nucleotide contribution scores for a 160-bp-long region within the IREN5 enhancer. The region is interpreted by models trained on astrocytes from healthy and injured cords. Motifs with higher contribution scores are highlighted (gray area) and the transcription factors recognizing such sequences are reported below. The coverage plot displays the observed accessibility of IREN5, along with the region displayed in the contribution plot. **d**, UMAP visualization of the astrocytes profiled with the AAV-based reporter assay and colored by injury condition. **e**, Feature plot displaying expression levels of *Gfap*. **f**, Feature plots reporting the read counts for each of the IRENs detected in the dataset, along with eHGT_380h, an astrocyte-specific human enhancer from Mich et al.^7^.

An exciting possibility that follows the identification and decoding of IRENs is their use to drive gene expression in specific cell states. We, thus, sought to determine whether we could achieve selective genetic targeting of reactive astrocytes at the injury site using systemically delivered adeno-associated viruses (AAVs), a therapeutically relevant gene delivery vector (Fig. 5a).

**Extended Data Fig. 7:**
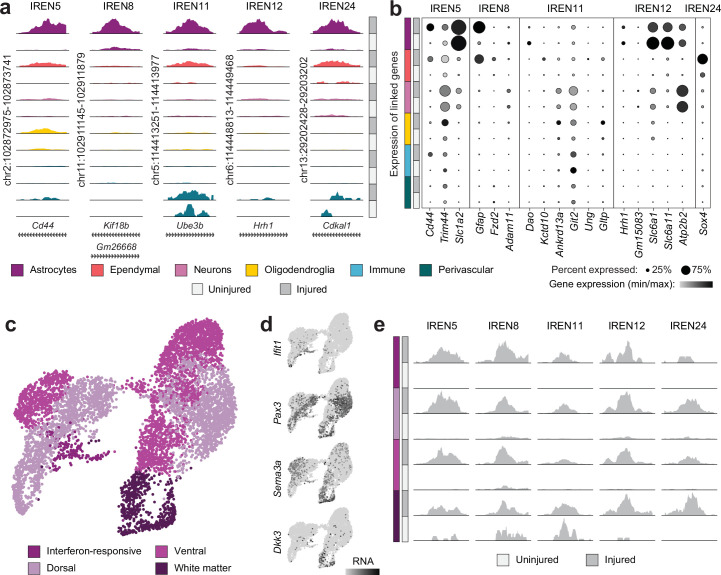
Accessibility and gene regulation of candidate IRENs across cell types. **a**, Coverage plots of the regulatory regions driving transgene expression in our AAV-based reporter assay. Tracks are colored by cell type and split by injury condition. **b**, Dotplot displaying the gene expression levels across cell types and injury condition, of genes linked to each of the IRENs reported in **a**. **c**, UMAP visualization of astrocyte subtypes. **d**, Feature plot displaying markers enriched in each astrocyte subtype. **e**. Coverage plot indicating the accessibility at the IRENs’ genomic coordinates, for each astrocyte subtype and injury condition.

We selected 19 candidate IRENs (566 to 1,188 bp) enriched in reactive astrocytes from the injured spinal cord that were linked to differentially expressed genes and that our models predicted to be accessible exclusively in this state (Fig. 5b, Extended Data Fig. 7a,b and Supplementary Table 1). Consistent with our previous observations, the model predicted that accessibility of these candidate enhancers was dictated by the presence of binding sites for the stimulus-induced AP-1 transcription factors along with astrocyte-specific factors (for example, SOX9 and NFI; Fig. 5c). To evaluate whether the IRENs were associated with astrocyte subtypes, we subclustered the astrocyte population and identified a recently described interferon-responsive cluster^12^ along with regionally distinct astrocytes^34,35^ (Extended Data Fig. 7c,d). All candidate IRENs exhibited accessibility across astrocyte subtypes (Extended Data Fig. 7e), indicating their potential role as pan-reactive astrocyte enhancers^36^.

**Extended Data Fig. 8:**
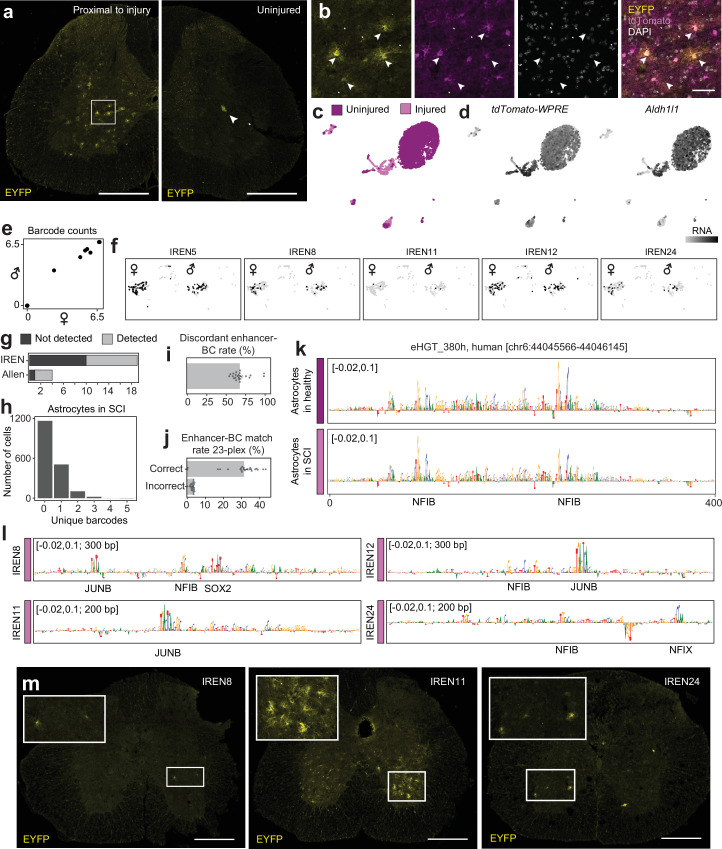
Overview of the AAV-based reporter assay. **a**, Overview of a spinal cord section collected from a Cx30-Tom reporter mouse injected with the AAV library and subjected to injury (left) and overview of an uninjured spinal cord collected from a Cx30-Tom reporter mouse injected with the AAV library (right). Scale bar: 250 μm. Data were collected from four mice. **b**, Magnification of the boxed area from **b** displaying tdTomato+ astrocytes labelled with EYFP. Scale bar: 50 μm. Data were collected from four mice. **c**, UMAP visualization of the scRNA-seq dataset obtained from the reporter assay. UMAP coordinates are calculated based on the gene expression profile and cells are colored according to sample of origin (that is, uninjured or injured spinal cord). **d**, Feature plots displaying expression levels of tdTomato-WPRE and *Aldh1l1* (that is, an astrocyte-specific gene). **e**, Scatterplot reporting the average read count for each of the injury-responsive enhancers (IRENs) detected in the injured dataset in each replicate (that is, Male and Female). **f**, Feature plots displaying the read counts for a few examples of IRENs detected in the injured dataset, split by replicate. **g**, Barplot displaying the number of barcodes delivered and detected in the scRNA-seq dataset corresponding to the IRENs and the cell type-specific (Allen) enhancers, respectively. **h**, Barplot reporting the frequency of detection for each unique barcode in each cell from the injured spinal cord. **i**, Barplots indicating the percentage of discordant enhancer-barcode pairs arising from pooled library packaging (n = 23). Data are presented as mean values and error bars represent the standard error of the mean. **j**, Barplots indicating the percentage of detected barcodes expected to correspond to correct enhancer-barcode pairs vs individual incorrect pairs in our 23-plex library. Data are presented as mean values and error bars represent the standard error of the mean. **k**, Nucleotide contribution scores, calculated by models trained on astrocytes from the uninjured and injured spinal cords, for the pan astrocyte enhancer eHGT_380h from^7^. Motif hits are indicated with the corresponding transcription factor binding such sequences. In brackets are reported the limits on the y-axis. **l**, Nucleotide contribution scores, calculated by models trained on astrocytes from the uninjured and injured spinal cords, for the IRENs validated in our data. Motif hits are indicated with the corresponding transcription factor binding such sequences. In brackets are reported the limits on the y-axis. **m**, Overview of spinal cord sections collected from mice subjected to single enhancer virus injection followed by injury. Sections were stained for EYFP. Scale bar: 250 μm. Data were collected from four mice.

To parallelize enhancer activity testing of candidate sequences, we developed a reporter assay based on AAV library delivery combined with single-cell RNA-seq profiling, which allowed us to simultaneously screen all candidate enhancers in vivo (Fig. 5a). This enabled us to test the ability of our putative IRENs, along with previously described astrocyte-specific enhancers^7^ as controls, to autonomously drive reporter expression in the right cell type and state. For this purpose, we sampled spinal cord tissue from mice that were systemically injected with the IREN AAV library and were subjected to an injury, as well as from uninjured controls (Fig. 5a). Consistent with most of the enhancers within the library gaining accessibility in reactive astrocytes (Extended Data Fig. 7a), we observed that the expression of enhanced yellow fluorescent protein (eYFP) was higher in regions proximal to the injury site than in uninjured tissue (Extended Data Fig. 8a,b). Further, eYFP expression was highly selective for astrocytes in animals that received the IREN library, suggesting a high degree of cell-type specificity (Extended Data Fig. 8a). Having confirmed the capacity of at least some enhancers within the library to drive injury-responsive reporter expression in astrocytes, we isolated astrocytes from these mice and performed single-cell RNA-seq with simultaneous barcode detection (Fig. 5c–f, Extended Data Fig. 8c,d and Methods). We detected barcodes belonging to 5 of 19 IRENs and 2 of 4 astrocyte-specific controls with high replicate concordance (Extended Data Fig. 8e–h). We note that although we used pooled AAV packaging, which led to an expected high degree of discordant enhancer–barcode pairs in the library (~60%; Extended Data Fig. 8i)^37^, in our 23-plex pool correct pairs had a tenfold higher chance of being detected than discordant ones (Extended Data Fig. 8j and Methods). All detected IRENs drove reporter expression exclusively in reactive astrocytes from the injured spinal cord (Fig. 5d–f), consistent with their pattern of accessibility. By contrast, an astrocyte-specific enhancer validated in previous studies^7^ labeled cells in both reactive and nonreactive states (Fig. 5f). Interestingly, the regulatory sequence of this enhancer of human origin carried transcription factor binding sites for NFIB, which were deemed important for cell-type specificity by our model trained on mouse astrocytes (Extended Data Fig. 8k). Unlike our astrocyte IRENs, the human sequence was not predicted by the reactive astrocyte model to encode functional AP-1 motifs (Fig. 5b,c and Extended Data Fig. 8l), in line with our predictions that this would confer injury-dependent activity (Extended Data Fig. 8m). This further suggests that regulatory principles for enhancer activity are conserved across species^38,39^.

Together, using a single-cell-resolved in vivo enhancer reporter assay, we have identified enhancer elements that can drive expression in a genetically defined cell state.

### IREN AAVs target reactive astrocytes across the CNS

**Fig. 6:**
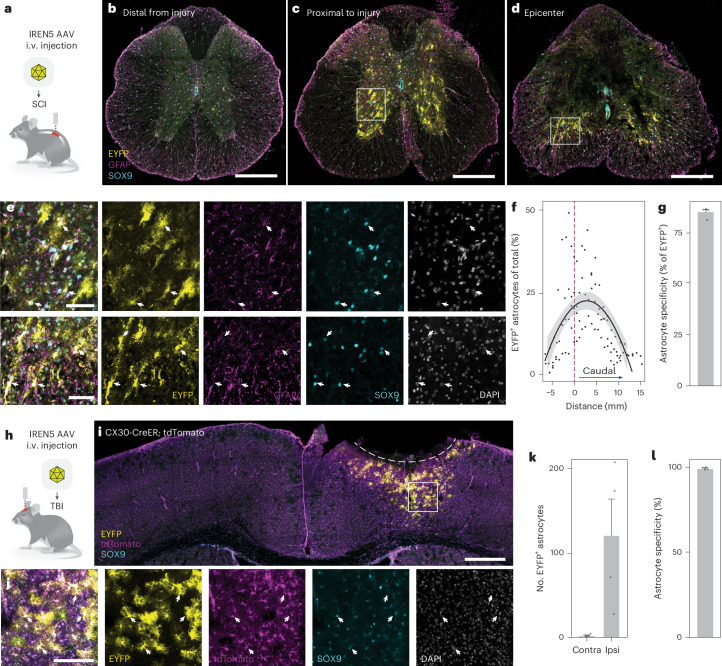
IREN5 AAV targets reactive astrocytes across the CNS. **a**, Schematic overview of the experimental design for the in vivo validation of IREN5-driven reporter expression in the spinal cord. The enhancer virus was intravenously injected in animals that were subjected to a spinal cord injury 14 days later. **b**–**d**, Overviews of spinal cord sections collected from the same mouse, which had been injected with the IREN5 enhancer virus, injured and analyzed 3 days after injury. The regions correspond to areas that are distal (**b**; ~5 mm rostral to the epicenter) or proximal (**c**) to the injury site or at the epicenter (**d**); scale bars, 250 μm. Data were collected from four mice. **e**, Magnification of the boxed areas from **c** displaying eYFP-labeled cells. Arrowheads point to examples of GFAP^+^SOX9^+^ eYFP-labeled astrocytes. Data were collected from four mice. **f**, Quantification of eYFP^+^ astrocytes in the spinal cord across the tissue segment spanning the injury (*n* = 4). The dashed line marks the epicenter of the lesion, and quantification was performed at different distances from the injury site (*x* axis). Measurements were obtained for four samples, and a quadratic function was fit to the data to display the spatial distribution. **g**, Bar plot reporting the average proportion of eYFP^+^ cells identified as astrocytes across the sections of spinal cord (*n* = 4). Data are presented as mean value, and error bar represents the standard error of the mean. **h**, Schematic overview of the experimental design for the in vivo validation of IREN5-driven reporter expression in the brain. The enhancer virus was intravenously injected in animals that were later subjected to a traumatic brain injury (TBI). **i**, Overview of the cortex at 3 days after injury from a mouse subjected to injury 14 days following enhancer virus injection. The dashed line emphasizes the lesion site; scale bar, 600 μm. **j**, Magnification of the boxed area from **i** displaying eYFP^+^ astrocytes. Arrowheads point to a few examples of tdTomato^+^SOX9^+^ eYFP-labeled astrocytes; scale bar, 150 μm. **k**, Bar plots reporting the number of eYFP^+^ astrocytes in the injured hemisphere compared to the contralateral (Contra) intact side (*n* = 4). Data are presented as mean values, and error bars represent the standard error of the mean; Ipsi, ipsilateral. **l**, Bar plots displaying the proportion of eYFP^+^ cells identified as astrocytes (*n* = 4). Data are presented as mean value, and the error bar represents the standard error of the mean.

**Extended Data Fig. 9:**
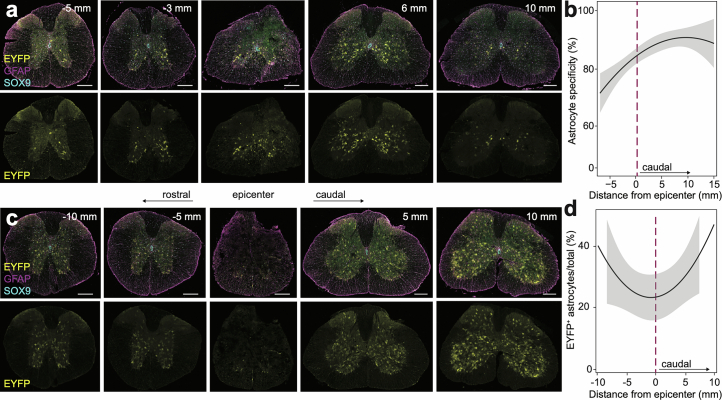
Cell labelling by IREN5 and pan-astrocyte eHGT_380h enhancer viruses in SCI. **a**, Overview of sections spanning the region around the injury site in the spinal cord sampled from a mouse injected with the injury-responsive IREN5 enhancer AAV. Sections are stained with antibodies against EYFP, GFAP, and SOX9. Scale bar: 200 μm. **b**, Plot representing the changes in astrocyte-specificity for the IREN5 enhancer across the spinal cord segment encompassing the injury (n = 4). Dashed line represents the epicenter of the lesion. **c**, Overview of sections spanning the region around the injury site in the spinal cord sampled from a mouse injected with the cell type-specific human enhancer AAV from^7^. Sections are stained with antibodies against EYFP, GFAP, and SOX9. Scale bar: 200 μm. **d**, Plot displaying the proportion of EYFP+ astrocytes across the spinal cord segment from c (n = 4). The number of labelled cells is normalized against the total number of SOX9-expressing cells on each tissue section. Dashed line represents the epicenter of the lesion.

To study the capacity of enhancers to drive expression in reactive astrocytes in preclinical models of CNS injury, we sought to individually validate all candidate IRENs from the in vivo screen. We delivered enhancer AAVs systemically before injury and detected astrocyte-specific signal 3 days after spinal cord injury from four out of five candidates (Extended Data Fig. 8m). We next focused on candidate IREN5, an enhancer linked to the pan-glial-responsive gene *Cd44* that had the highest labeling efficiency (Fig. 6a–d). In line with our reporter assay, we did not detect eYFP^+^ astrocytes in regions distal from the injury site (Extended Data Fig. 9a). Importantly, astrocytes close to and at the lesion border were efficiently and specifically labeled (85%; Fig. 6e–g and Extended Data Fig. 9b), demonstrating the ability of IREN5 to autonomously drive gene expression with spatiotemporal precision in reactive astrocytes. By contrast, and consistent with our AAV library screen, the astrocyte-specific human enhancer^7^ drove eYFP expression in astrocytes across the spinal cord (Extended Data Fig. 9c). Interestingly, activity of this enhancer mildly decreased in proximity to the lesion (Extended Data Fig. 9d), contrary to the pattern observed for the IREN (Fig. 6f).

**Extended Data Fig. 10:**
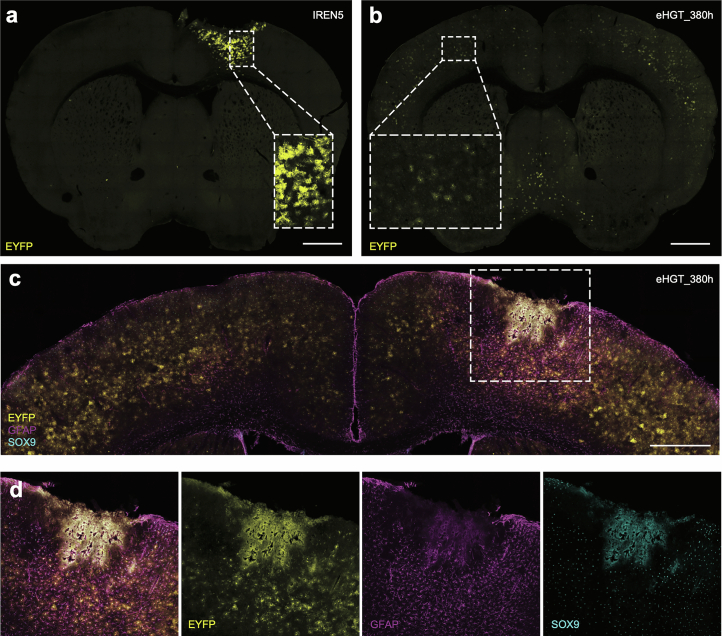
Cell labelling by IREN5 and pan-astrocyte eHGT_380h enhancer AAVs in TBI. **a**, Overview of the brain of a mouse systemically injected with IREN5 and subsequently subjected to a traumatic brain injury. Sections are stained with antibodies against EYFP. Scale bar: 1000 μm. Data were collected from four mice. **b**, Overview of the brain of a mouse systemically injected with the pan-astrocyte enhancer virus (eHGT_380h from^7^). Sections were stained with antibodies against EYFP. Scale bar: 1000 μm. Data were collected from four mice. **c**, Overview of the brain of a mouse systemically injected with the pan-astrocyte enhancer virus and subjected to a traumatic brain injury). Sections are stained with antibodies against EYFP, GFAP, and SOX9. Scale bar: 1000 μm. Data were collected from four mice. **d**, Magnification of the injury area from **c**.

We next explored whether the ability of our identified enhancers to target reactive astrocytes would generalize to other CNS injuries. To this end, we injected IREN5 reporter AAVs intravenously into mice that were then subjected to stab wound traumatic brain injury in the motor cortex to study reporter expression 3 days after injury (Fig. 6h). In this model, although the pan-astrocyte-specific enhancer labeled astrocytes throughout the brain, IREN5 drove eYFP expression exclusively in reactive astrocytes surrounding the injured cortex (Fig. 6i,j and Extended Data Fig. 10). By contrast, astrocytes in the unaffected contralateral hemisphere, as well as other brain regions, including the borders, remain unlabeled (Fig. 6i–k). Reactive astrocyte targeting was efficient, with a large fraction labeled across the entire lesion border with high specificity (98%; Fig. 6l).

Together, we have identified IREN elements that enable specific targeting of reactive astrocytes across the CNS using therapeutically relevant viral vectors following systemic administration.

### Sequence determinants of injury-induced enhancer activity and specificity

**Fig. 7:**
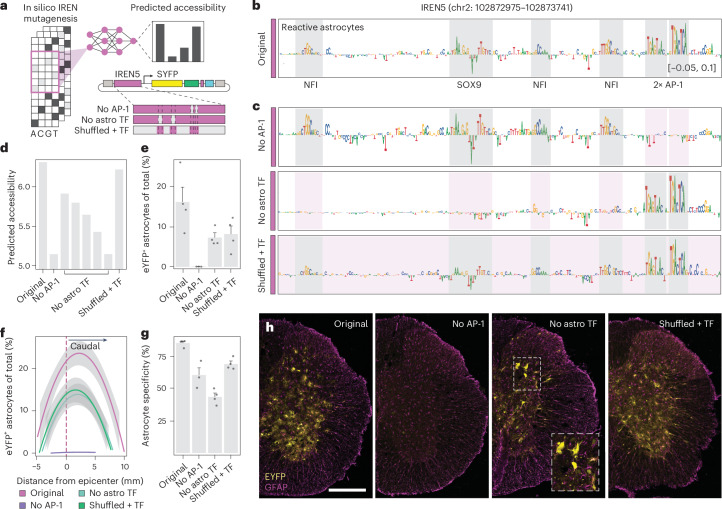
Sequence determinants of IREN5 specificity. **a**, Schematic representation of synthetic enhancer validation. IREN5 DNA sequence was mutated in silico to ablate binding sites for AP-1 or astrocyte-specific transcription factors. The reactive astrocyte model was then used to predict activity of the synthetic sequences. Enhancer viruses carrying the mutated versions of IREN5 were produced and used in combination with a spinal cord injury to experimentally validate the sequences’ labeling performance; astro, astrocyte; TF, transcription factor. **b**, Nucleotide contribution scores for IREN5, as interpreted by the reactive astrocyte model. Motifs with higher contribution scores and recognized by astrocyte-specific and AP-1 transcription factors are highlighted in gray. **c**, Mutated IREN5 sequences for which AP-1 (top) or astrocyte-specific sites (middle) were ablated. The bottom plot represents a synthetic sequence for which all nucleotides had been shuffled, with the exception of the recognized transcription factor binding sites, which remained constant. **d**, Bar plots reporting the accessibility predicted for each of the synthetic sequences by the reactive astrocyte model. **e**, Bar plots displaying the labeling performance of the synthetic sequence, as recorded in the experimental validation (*n* = 3 for no AP-1 and *n* = 4 for the other conditions). **f**, Plot displaying labeling efficiency as a function of distance from the injury epicenter. The dashed line represents the epicenter of the lesion. Curves are split based on the enhancer virus tested (that is, based on the original IREN5 sequence or the mutated versions). **g**, Bar plot reporting the labeling specificity for each synthetic sequence (*n* = 3 for no AP-1 and *n* = 4 for the other conditions). **h**, Overview of spinal cords from mice subjected to injury following systemic injection of the enhancer viruses; scale bar, 250 μm.

Having established the use of IREN5 for reactive astrocyte targeting, we explored whether its activity was dependent on the general enhancer syntax learned by the model. We first leveraged the interpretability of our models to establish sequence-to-function relationships through in silico mutagenesis (Fig. 7a). Our reactive astrocyte model predicted an architecture composed of tandem AP-1 motifs along with motifs for the lineage-specific factors SOX9 and NFI (Fig. 7b). We then predicted the accessibility when ablating AP-1 motifs, lineage-specific motifs or a synthetic sequence that shuffles the original sequence but preserves the learned motif syntax (Fig. 7a). Ablation of AP-1 led to a drastic reduction in predicted accessibility in reactive astrocytes (despite preserving >97% of the enhancer sequence), whereas ablation of SOX9 and NFI motifs led to a more gradual reduction. Interestingly, the shuffled synthetic version had a predicted accessibility that recapitulated the original accessibility better than the individual single motif mutants despite having mutations in >90% of the original sequence (Fig. 7c,d). Finally, we aimed to validate the model predictions in vivo by delivering synthetic enhancer mutant AAVs systemically before injury and measure reporter signal 3 days after spinal cord injury. In agreement with the model, ablation of the injury-responsive AP-1 module led to a drastic reduction in reporter activity, demonstrating a strong AP-1 dependency on IREN5 commissioning (Fig. 7e,f). In turn, ablation of the SOX9 and NFI sites led to a milder decrease in reactive astrocyte labeling but was accompanied by a drastic drop in specificity (Fig. 7g), supporting a role for cooperative binding with AP-1 in cell-state enhancer selection. Interestingly, the synthetic version best recapitulated reactive astrocyte targeting efficiency and specificity (Fig. 7f–h).

Together, our in vivo reporter assays validate that IREN activity and specificity are encoded by a cooperative syntax that involves injury-induced AP-1 factors and lineage-specific transcription factors.

## Discussion

We have identified and characterized cell-type-specific IRENs in the CNS. Previous studies have focused on the identification and characterization of enhancers specific to cell types that have enabled genetic access to specific neuronal and glial cell populations using viral vectors^6–8^. In parallel, other studies have identified enhancer elements active after tissue damage in multiple tissues, but these have frequently lacked cell-type resolution^5,40,41^. Here, we discovered that the genome contains a large number of regulatory sequences whose activity is not only cell-type specific but also injury responsive.

Using deep learning models, we decoded the enhancer syntax underlying injury-responsive gene regulation, successfully identifying sequence features that predict enhancer accessibility in distinct cell states. These models revealed that AP-1 family transcription factors play a critical role in injury-induced enhancer commissioning. Injury-responsive AP-1 activation is a widespread phenomenon that has been described in glial cells from the central and peripheral nervous system. Studies in fibroblasts, macrophages and epithelial stem cells have suggested that AP-1 interaction partners, including SOX9, are involved in enhancer selection after stimulation^42–44^. Here, we find that the DNA sequence of injury-responsive enhancers directs AP-1 interactions with lineage-specific factors to encode enhancer selection across glial cells and ultimately the cell-type specificity of the injury response program. This DNA-guided transcription factor cooperation, likely facilitated by transcription factor interactions, offers an efficient encoding mechanism to direct cell-type-specific gene expression programs following injury without the need for specific injury-induced factors. Different glial cells, however, relied on this mechanism at varying degrees, with microglia commissioning injury-induced enhancers through more diverse transcriptional effectors. It is likely that the larger diversity of regulatory mechanisms reflects the heterogeneity of states that microglia acquire after injury^45^, and future modeling at increasingly higher cell-state granularity may decode additional DNA-guided cooperativity for other families of transcription factors^46^.

The decoding of these sequences allowed us to specifically and conditionally target genetically defined reactive cell states within the injured CNS, particularly focusing on reactive astrocytes. To identify candidate enhancers, we relied on in vivo parallel enhancer screening, from which 20–25% of candidates showed state-specific reporter activity. This is not far from the reported 30% on target validation for cell-type-specific cortical enhancers^47^. However, given that our deep learning models predicted local accessibility, and not transcriptional activity, it is likely that expression or activity modeling may allow an increase in positive enhancers, critical for larger-scale screening. In addition, we focused on strong pan-reactive astrocyte enhancers, but dual-barcode screening designs with shorter homology regions may allow the recovery of weak or subtype-specific enhancers^48,49^.

Reactive astrocytes are common to multiple neurological pathologies, including spinal cord injury, traumatic brain injury, stroke and neurodegenerative diseases^12,32,33^. Thus, our findings on the IRENs associated with reactive astrocytes are likely applicable to a broader range of conditions. Similarly, recent studies have identified reactive states for other glial cells, such as microglia, in response to injury and neuroinflammation^50^. Our approach lays the groundwork for targeting these and other reactive cell states via the identification of specific IREN elements in combination with novel engineered viral vectors.

In summary, our decoding of the regulatory logic and design principles of IRENs not only advances our understanding of gene regulation but also enables the design of sequences that can be programmed to selectively target disease-related cell states for therapeutic applications.

## Methods

### Animals

Experimental procedures were performed in compliance with governmental and ethical specifications approved by the Karolinska Institute (Stockholm, Sweden) and the Stockholms Norra Djurförsöksetiska Nämnd. Animals used in experiments were housed in individually ventilated cages containing two to five mice per cage. They were maintained on a 12-h light/12-h dark cycle and provided water and food ad libitum. Wild-type C57BL/6 mice were obtained from Janvier, whereas transgenic mice were obtained by crossing *Foxj1*-CreERT2 (ref. ^14^) or *Cx30* (ref. ^51^) with *R26R*-tdTomato Cre reporter mice^15^. Animals entered the study at the age of 8–12 weeks and were assigned to each experimental condition in a balanced design. Each condition, furthermore, encompassed both female and male mice.

Induction of genetic recombination was achieved through oral administration of tamoxifen (Sigma; 20 mg ml^−1^ in 1:9 ethanol:corn oil solution) at 2 mg per injection on two consecutive days. Subsequent procedures were performed at least 2 weeks after the last injection to ensure expression of the fluorescent reporter and to avoid potential confounders from circulating tamoxifen^52^.

### Spinal cord injury

Mice were deeply anesthetized with isoflurane (4% at 400 ml min^−1^ air for induction, 2% for maintenance) and kept on a heated pad throughout the procedure. They received subcutaneous injections of analgesics (buprenorphine, Schering-Plough, 0.1 mg per kg (body weight) and carprofen, Pfizer, 5 mg per kg (body weight)) and antibiotics (sulphadizine/trimethoprim, Tribrissen vet., MSD, 100 mg per kg (body weight)) in 0.5 ml of saline.

Contusion injury was performed at T9 using the Infinite Horizon Impactor (IH-0400, PSI) equipped with a 1.3-mm tip. A laminectomy was performed at the T8–T9 level to expose the dorsal portion of the spinal cord, while the dura mater was left intact. The vertebral column was stabilized with clamps (PSI) fixed on the foramina of T8 and T10. Local anesthetic (xylocaine, 10 mg ml^−1^) was applied topically on the spinal cord surface (two drops) before the impact. A force of 55 kDyne was used, and the injury was visually confirmed by the occurrence of a bruise on the dorsal portion of the spinal cord. Animals for which the displacement was below 350 were excluded from the experiment. After the contusion, the muscle overlaying the laminectomy and the opening on the skin were sutured with nonabsorbable and absorbable stitches, respectively. Animals were allowed to regain consciousness in a heated cage before being transferred to their original cage, now equipped with an elevated floor grid.

During the first three postoperative days, animals received antibiotic treatment and analgesia (buprenorphine, Schering-Plough, 0.1 mg per kg (body weight) twice per day and carprofen, Pfizer, 5 mg per kg (body weight) once per day). Their diet was supplemented with high-energy nutritional food (DietGel Boost, Clear H2O), and animal weight was monitored daily for the first week and then weekly until the experimental endpoint. Animals with a weight loss of >15% of the preoperative weight were killed. Bladders were expressed twice daily until the animals regained control.

### Stab wound injury

Animals were deeply anesthetized with isoflurane and kept on a heated pad throughout the procedure. The head was fixed onto a three-point stereotactic frame, and an incision (5 mm) was performed on the skin to expose the skull. An opening was made in the skull at +2.5 mm medio–lateral and +1.0 mm rostral from bregma. A syringe with a 26-gauge blunt needle fixed onto the stereotactic apparatus was lowered to 0.7 mm below the dura surface. To extend the lesioned area, the needle was moved 1 mm along the rostro–caudal axis. After the procedure, animals were transferred to a heated cage until they had regained consciousness and then moved back to their home cage.

### Retro-orbital injection

Mice were deeply anesthetized with isoflurane before intravenous injection. Insulin syringes (0.5 ml with a 30-gauge needle) were loaded with the virus (see below) diluted in saline (50 μl per injection). The injection was performed unilaterally into the retrobulbar sinus after covering the eye with ophthalmic ointment. The needle was adjusted to an angle of 30° and had the bevel facing upward. After the procedure, animals were allowed to regain consciousness in a heated cage before being transferred back to their home cage.

### Tissue preparation for multiomics

Mice were killed at 1, 3, 7 and 28 d.p.i. or in the absence of an injury as controls. They were deeply anesthetized with an intraperitoneal injection of sodium pentobarbital (100 μl per injection) and transcardially perfused with sterile HBSS (without calcium and magnesium). Fresh spinal cords were immediately microdissected and cleared of the meninges. We obtained 5-mm tissue segments around the injury epicenter or at the same rostro–caudal level in uninjured animals. Spinal cords from wild-type C57BL/6 mice were dissociated and processed to isolate nuclei. We pooled two samples per experimental time point (that is, one tissue sample per sex) and used a Chromium Nuclei Isolation kit (10x Genomics, PN-1000493) to prepare suspensions of single nuclei. These were, subsequently, stained with fluorescently conjugated antibodies to NEUN (1:500; Millipore, MAB377) or SOX10 (1:200; Millipore, AB5727) for 1 h on ice and with 7-AAD (1:50) before sorting on a BD FACS Influx sorter with a 100-μm nozzle.

We used the transgenic line Foxj1–tdTomato to enrich for ependymal cells. For this experiment, we pooled together four cords per experimental condition (that is, two animals per sex) and enzymatically digested the tissue to obtain single-cell suspensions. Briefly, we used papain as the digestion enzyme and a GentleMacs Dissociator (Miltenyi) to triturate the samples according to specifications provided in the Miltenyi’s Adult Brain Dissociation protocol. Whole cells were sorted based on expression of the fluorescent reporters and collected in sorting buffer containing 1% bovine serum albumin for subsequent nuclei isolation (performed according to the 10x protocol CG000124 Rev F). Sequencing-ready libraries were prepared following the Chromium Next GEM Single Cell Multiome protocol (10x Genomics, CG000338 Rev F) and sequenced in an Illumina NextSeq or an MGI machine.

### Tissue preparation for immunostaining

Mice were deeply anesthetized and transcardially perfused with PBS, followed by 4% paraformaldehyde (Sigma) in PBS. Vertebral columns were isolated and postfixed overnight in 4% paraformaldehyde at 4 °C. Subsequently, the tissue was extracted, rid of the meningeal layers and placed in 30% sucrose at 4 °C. We obtained 5-mm segments centered around the injury and embedded them in OCT. The samples were sliced into 20-μm-thick coronal sections using a Cryostar NX70 (Thermo) and stored at –20 °C until further processing. Brains of animals subjected to a stab wound injury were extracted after perfusion and fixed in 4% paraformaldehyde. They were then sectioned using a vibratome into 50-μm-thick coronal sections.

### Immunostaining and quantification

Cryosections were equilibrated to room temperature and incubated in blocking buffer (10% donkey serum and 0.3% Triton X-100 in DPBS) for 1 h. Primary antibody incubation was performed overnight in a humidified chamber at 4 °C. We used the following primary antibodies diluted in blocking buffer: chicken anti-GFP (1:1,000; Aves, GFP1010), rabbit anti-RFP (1:1,000; Rockland, 600-401-379), rabbit anti-GFAP (1:500; Dako, Z0334) and goat anti-SOX9 (1:250; R&D, AF3075). Sections were subsequently washed (three times for 10 min each) in DPBS and incubated with fluorescently conjugated secondary antibodies (Alexa Fluor 488, Alexa Fluor 555 or Alexa Fluor 647; 1:500, donkey, Thermo). Finally, sections were washed three times for 10 min each in DPBS. DAPI was included at the last washing step (1 μg ml^−1^; BD). Brain sections were stained as free-floating sections using the same antibodies and concentrations. Images were acquired in a Zeiss LSM 700 confocal microscope and processed using Fiji.

eYFP^+^ cells were manually counted in a spinal cord segment spanning 5 mm rostral and 10 mm caudal to the injury epicenter. The total number of labeled astrocytes per section was normalized by the number of SOX9^+^ cells. SOX9 quantifications were performed with the automated Analyze Particle tool in Fiji after binarizing the images. For the stab wound injury model, four sections spanning the injury site were used for quantifications for each animal. The contralateral hemisphere on the same section was used as a control.

### Raw sequencing data processing

Raw reads were processed using the CellRanger-ARC pipeline (v2.0.2, 10x Genomics) and mapped to the mm10 reference genome (mm10, v2.0.0 from Cell Ranger) supplemented with tdTomato DNA sequence. Raw reads from the MGI sequencing runs were obtained in fastq format, with R2 and the indexes in a single R2 file. For demultiplexing, we first split the R2 file and reverse-complemented the index reads using a custom script (see Code availability) and demultiplexed by sample using deML (v1.1.4). Subsequently, the cellranger-arc count function was used to map data to the reference genome. Multimodal data were first preprocessed and filtered separately by sample and by modality before integration for downstream analysis. The experiment yielded a total of 21 high-quality samples (Supplementary Fig. 1).

### Processing of gene expression data

Gene expression data from each sample were processed individually to remove ambient RNA and identify low-quality cells and putative doublets. CellBender (v0.3.2) was used on raw, unfiltered gene expression matrices to identify and remove ambient RNA. We used default parameters and a false positive rate of 0.05 to correct the matrices. Raw matrices were still used to identify poor-quality nuclei and doublets, whereas CellBender-corrected transcriptional data were used for all downstream multiomics analyses. Raw gene expression data were scaled to a constant factor of 10,000, normalized for sequencing depth and log-transformed using Seurat R package^53^ (v4.3.0). Dimensionality reduction was then performed with principal component analysis, and the top 15 components were used to organize nuclei in the UMAP space.

### Processing of chromatin accessibility data

Fragment files from each sample were merged into a common dataset to perform peak calling using ArchR^54^ (v1.0.2). Specifically, we ran addIterativeLSI on the TileMatrix with default parameters for dimensionality reduction, followed by harmony integration (computed with addHarmony() function), addUMAP() and addCluster() at a resolution of 1. The merged data were used for cluster-wise peak calling using addReproduciblePeakSet() with default parameters. This consensus peak set was, then, imported into Signac’s^55^ (v1.12.0) workflow to recalculate feature matrices and create new chromatin assay objects using FeatureMatrix() followed by CreateChromatinAssay(). This was done individually, for each sample in the dataset, and was followed by sample-wise dimensionality reduction with RunTFIDF(), RunSVD and RunUMAP (on dimensions 2 to 15). Gene annotations from EnsDb.Mmusculus.v79 (v2.99.0) were used to annotate genomic regions in the chromatin data and for computing TSS enrichment scores.

### Filtering of multiomic data

We discarded nuclei with fewer than 200 unique molecular identifiers (UMIs) and 300 fragments in the RNA-seq and ATAC-seq assays, respectively. Subsequently, we inspected the number of features and UMIs in a cluster-wise approach, which accounts for differences in transcriptional activity across neuronal and non-neuronal cell types. We removed observations that were over 3 × interquartile range for the (1) UMI count, (2) number of unique genes, (3) number of fragments, (4) percentage of mitochondrial content, (5) TSS enrichment and (6) nucleosome signal.

Next, we removed putative doublets from each modality separately, removing observations identified as doublets in either assay. For the gene expression data, we used DoubletFinder^56^ (v2.0.3) on Seurat’s computed principal components (1 to 15) and cluster identities. First, we ran parameter sweep to obtain the best pK value, which was then used together with a pN of 0.25 to compute doublet likelihood scores. The expected doublet rate was adjusted sample-wise based on the size of the dataset (that is, following a linear increase between the number of observations and number of doublets). In the chromatin data, doublets were identified with the scDblFinder package^57^ (v1.16.0) using 1,000 features, LSI dimensions 2 to 15, and an expected doublet rate adjusted to the number of observations per sample. Furthermore, a few more potential doublets were manually filtered out after integration across all samples, based on the occurrence of incompatible gene expression profiles.

### Integration of multiomic data

Clean, high-quality data from each sample were merged into a unique multimodal dataset encompassing approximately 67,000 nuclei (Fig. 1). Dimensionality reduction was recomputed for each modality using PCA and LSI on the top variable features (‘nfeatures = 2,000’ for RNA and ‘min.cutoff = 10’ for ATAC). Next, the top 30 components (excluding the first component for the ATAC assay) were used to calculate multimodal nearest neighbors and the UMAP visualization and to cluster data into major cell types (that is, achieved using a resolution of 0.1). Furthermore, we computed UMAP and clustering for each modality separately and compared the granularity of the data at a higher clustering resolution (that is, 1.1; Fig. 1).

We used Signac’s LinkPeaks() function to link genes and accessible regions in the whole dataset, as well as the cell-type-specific objects. This allowed us to obtain linkages based on correlation between transcriptional activity and chromatin accessibility, even for smaller cell populations.

### Differential analysis

We used Seurat’s FindAllMarkers() or FindMarkers() to identify differentially expressed genes and differentially accessible regions across cell types or across experimental conditions. Wilcoxon’s test statistics were used to identify significantly enriched features at a *P* value of <0.05 and average log_2_ (FC) of > 0.5. Differential features identified between uninjured and injured conditions were, furthermore, clustered based on their dynamic profile across time points using TCseq (v1.28.0) fuzzy clustering.

To gain functional insight into the gene regulatory programs characterizing the cellular response to spinal cord injury, we ran GO analysis on differentially expressed genes using gprofiler2 (ref. ^58^; v0.2.3) and recorded the top 10 GO terms for ‘Biological Process’, excluding general processes encompassing more than 2,000 genes. Differentially accessible regions were further investigated using motif enrichment analysis (that is, Signac’s FindMotifs()).

### Machine learning model architecture

Our deep neural network models were trained and interpreted using the ChromBPNet pipeline (v0.1.7), following recommendations outlined in Nair and colleagues^22^ and the code provided at https://github.com/kundajelab/chrombpnet. All analyses were performed with Tensorflow (v2.8.0) and Keras (v2.8.0). The architecture of the models used in this paper is derived from ChromBPNet, which is, in turn, based on BPNet^18^. Briefly, the models take one-hot encoded DNA sequences of 2,114 bp as input, which is passed onto an initial convolutional layer composed of 512 filters, kernel size of 21, padding = ‘valid’ and activation = ‘relu’. This is followed by 9 dilated convolutions characterized by 512 filters, kernel size of 3, padding = ‘valid’, activation = ‘relu’ and dilation rate of 2^layer_number^. The output of the final layer is used to generate (1) profile log-likelihoods through an additional convolution with a kernel size of 75, followed by a flatten layer, and (2) count predictions through global average pooling and a dense layer. Adam optimizer was used with a learning rate of 0.001, and early stopping was implemented after five epochs without improvement in validation loss.

The Tn5 bias model was trained with ChromBPNet pipeline bias function on a subset of our data encompassing one fragment file from each experimental condition (that is, injury time point and enrichment strategy). We used peaks in chromosomes 8 and 19 for validation, chromosomes 1 and 3 for testing and the rest for training.

### Processing input data for cell-type model training

Using our chromatin accessibility data and the Tn5 bias model, we trained models at the cell-type level. To do this, we first created pseudobulks using Signac’s SplitFragments(). Nonpeak region lists were generated for each set of peaks using chrombpnet’s prep nonpeaks function. Sequences were shifted by +4 bp on the ‘+’ strand and by –5 bp on the ‘–’ strand to adjust for the Tn5 offset. We performed data augmentation by allowing 500 bp of jittering, as well as performing reverse complement for a random sample of sequences during training. For cross-validation, we prepared five combinations of training, test and validation sets, using peaks on 16 chromosomes for training, and 2 for test and validation (see Code availability to access the splits used for cross-validation).

### Motif analysis

To learn the importance of sequences of interest at base-pair resolution, we computed nucleotide contribution scores using the DeepLIFT algorithm (v.0.6.13.0) with the DeepSHAP implementation^24^. TF-MoDISco^25^ (https://github.com/jmschrei/tfmodisco-lite, v2.0.7) was then run with a maximum of 1 million seqlets to extract motifs, which were then matched against a motif database^59^. We retained transcription factors matching each motif and expressed in at least 1% of cells within the cluster of interest. We also ran conventional motif enrichment analysis on differentially accessible regions using Signac’s AddMotifs() and the ‘CORE’ vertebrate collection in JASPAR2020 database.

### Gene regulatory network inference

The SCENIC+^31^ workflow (v1.0a1) was performed following the instructions outlined at https://scenicplus.readthedocs.io. A consensus peak set was defined from pseudobulked fragments (that is, split by cell type and time point) with pseudobulk_peak_calling.peak_calling and iterative_peak_calling.get_consensus_peaks from pycisTopic (v2.0a0), using default parameters. Latent Dirichlet allocation (LDA) modeling was conducted with the lda_models.run_cgs_models_mallet function to identify 15 topics. Regions were then binarized by topic using both the top-3000-peaks method and Otsu’s threshold, and cells were binarized per topic via Li’s method. Accessibility was imputed (scale_factor = 10^6^), and highly variable regions were identified (min_disp = 0.05, min_mean = 0.0125, max_mean = 3, 20 bins). Differentially accessible regions were then called (adjusted *P* < 0.05, log_2_ (FC) > log_2_ (1.5)), and gene activity scores were calculated using a 100-kb upstream/downstream search space.

In SCENIC+, metacells (*n* = 10) were generated to stabilize coaccessibility estimates, and peaks were linked to genes based on correlation of signals and a ±150-kb search space. Motif over-representation in candidate enhancers was compared to background (false discovery rate < 0.05, minimum log_2_ (FC) = 1 and mean foreground threshold of 0). Motifs required an area under the curve of >0.005 and a normalized enrichment score of >3.0 to be retained. Transcription factor–gene and region–gene links were inferred using a stochastic gradient boosting machine (GBM) framework. Region–gene correlations were computed alongside importance scores from GBM; only the top 5–15 regions per gene (quantiles 0.85, 0.90 and 0.95) were retained, and a minimum of 0 regions per gene was allowed. Regions were ranked by GBM importance, and edges with a correlation of <0.05 were discarded. Regulon enrichment (gene set enrichment analysis) was run with 1,000 permutations to refine transcription factor–target relationships. After motif enrichment and edge selection, candidate region–gene and transcription factor–gene edges were merged to form enhancer-driven regulons.

### CUT&Tag

First, frozen tissues were collected and processed for nuclei isolation and sorting as described above. In total, 150,000 nuclei from each sample were used to perform CUT&Tag, as previously described^60^, with the following modifications for bulk processing. Nuclei were incubated overnight at 4 °C with anti-SOX9 (1:100 dilution; Sigma, AB5535) in antibody buffer. Twenty percent of the nuclei per sample were set aside as secondary-only controls and underwent all other steps in parallel. After washing, nuclei were incubated for 1 h with guinea pig anti-rabbit secondary antibody (1:50 dilution; Novus Biologicals, NBP1-72763) in DIG wash buffer. After washing, samples were incubated with loaded pA-Tn5 transposase (1:100 dilution; C01070001) and tagmented for 1 h at 37 °C. Next, DNA was purified using a MinElute Reaction cleanup kit (Qiagen, 28204) and directly used for PCR amplification and indexing using Ad1.NoMx and Ad2.N oligonucleotides^61,62^ for 15 cycles using NEBnext High Fidelity master mix (New England Biolabs, M0541). Libraries were quantified using a Qubit dsDNA HS assay kit before sequencing. Samples were sequenced on an MGI G99 sequencer using paired-end reads according to the manufacturer’s protocols. Raw reads were demultiplexed by sample using deML (v1.1.4) and mapped to the reference genome with bowtie2 (2.5.4) with the following options: ‘–local –very-sensitive –no-mixed –no-discordant –phred33 -I 10 -X 700’. Duplicates were removed with picard (3.4.0), and peaks were called using SEACR (1.3) and ‘norm stringent’ options.

### In vivo enhancer reporter assay

Each enhancer was cloned into a CN2249-rAAV-eHGT_451m-minBglobin-SYFP2-WPRE3-BGHpA (Addgene, 164452) plasmid backbone in two steps. First, a barcoded plasmid library was made using a 200-bp ultramer containing two 12-bp variable regions flanking a 20-bp constant region, which was cloned into an XhoI-digested backbone (Supplementary Table 1) through NEBuilder HiFi DNA Assembly (New England Biolabs, E2621L). The construct was then transformed into Endura Electrocompetent cells (Biosearch Technologies, 60242-1) and spread onto a 245 × 245 mm LB agar (Duchefa Biochemie, L1706)–100 mg ml^−1^ ampicillin (Merck, A5354-10ml) dish for overnight incubation, after which the barcoded plasmid library was extracted through Qiagen Plasmid Plus Midi Prep. Second, the enhancers were extracted from mouse genomic DNA using overhang PCR (Supplementary Table 1) for assembly into an MluI/SacI-digested barcode library through Gibson assembly (New England Biolabs, E2611L), immediately followed by transformation into NEB Stable Competent *Escherichia coli* cells (New England Biolabs, C3040H) and overnight incubation. Individual colonies were incubated in 5 ml of LB broth (Gibco, 10855001) supplemented with 100 mg ml^−1^ ampicillin (Merck, A5354-10ml) and incubated overnight, after which the plasmids were recovered using a QIAprep Spin Miniprep kit (Qiagen). Both steps were verified using Sanger sequencing with primers EGFP-C and EGFP-N, respectively (Supplementary Table 1). All enhancer sequences included in the library are listed in Supplementary Table 1. Equimolar mixtures of all barcoded enhancer vectors were pooled and used for packaging into AAV-PHP.eB particles (AAVnerGene). We delivered the AAV library (2 × 10^11^ viral genomes) intravenously in mice carrying fluorescently labeled astrocytes (Cx30–Tom). We then subjected the mice to spinal cord injury and sampled labeled cells at 3 d.p.i., as well as from uninjured controls.

To read out the enhancer activity, sorted cells were processed using Chromium Single Cell 3’ v3.1 (10x Genomics, PN-1000121). To enrich the barcoded transcripts, after cDNA amplification, 25% of the cDNA was used for linear amplification with oEL197 (30 cycles). Next, barcode fragments were further amplified using oEL196 with Partial R1 for 15 cycles and oEL195 with Partial R1 for 4 cycles. These amplicons were then barcoded using Dual Index Plate TT SetA oligonucleotides (4 cycles) and sequenced on an MGI G99 sequencer. Sequencing reads were processed using CellRanger (v8.0.1) with the antibody capture pipeline adapted to detect the custom eYFP enhancer-associated barcodes (Supplementary Table 1). Count matrices including each barcode were processed using Seurat. Cells were filtered based on the count number (500 < counts < 50,000), UMAP was computed on the top 15 principal components, and astrocytes were identified based on canonical markers (*Aldh1l1*) and selected for downstream analysis.

### In vivo validation of endogenous and synthetic enhancer viruses

IREN5 was selected for individual validation based on the performance recorded in the reporter assay. In silico mutagenesis was applied to the endogenous DNA sequence to ablate sequence motifs. Tangermeme’s (v0.4.0) ersatz.dinucleotide shuffle function was used to shuffle the nucleotides, allowing for ablation of the motif while retaining similar GC content. Enhancer mutants and synthetic sequences were ordered as gBlocks (IDT) and cloned via Gibson assembly into the MluI/SacI-digested backbone as described above. Sequences used for the experimental validation are reported in Supplementary Table 5.

### Statistics and reproducibility

No sample size calculations were performed for this study, but the appropriate number of animals to allocate for each experimental condition was estimated based on previous studies using the same technologies. For the sequencing experiments, two to four animals were pooled for each condition. For the in vivo validation, three to four animals per condition were used. Mice were randomly allocated to experimental conditions, and each condition included equal numbers of females and males. For reproducibility of the multiomic analysis, data were processed using different computational tools, for instance, to identify peaks in the chromatin accessibility data (that is, MACS2, Signac, ArchR). Results were compared and confirmed to have good overlap. Whenever possible, quantifications were performed blinded to experimental condition. For the sequencing analysis, tools allowing unsupervised clustering were used (for example, Seurat).

### Data visualization

Most graphs for the multiomic analysis were generated using Seurat’s plotting functions or ggplot2 (3.4.4). Heat maps and hierarchical clustering of cell (sub)types and features were produced using pheatmap R package (v1.0.12). *Z* scores were used for the heat maps, whereas log-transformed data were used in the feature plots and violin plots. Circos plots were produced using differentially expressed genes (log (FC) > 1) and the circlize package^63^ (v0.4.16).

### Reporting summary

Further information on research design is available in the Nature Portfolio Reporting Summary linked to this article.

## Online content

Any methods, additional references, Nature Portfolio reporting summaries, source data, extended data, supplementary information, acknowledgements, peer review information; details of author contributions and competing interests; and statements of data and code availability are available at 10.1038/s41593-025-02131-w.

## Supplementary information

Supplementary Information Supplementary Figs. 1–5.

Reporting Summary

Supplementary Table 1 List of oligonucleotides used for library construction for the enhancer AAV experiment.

Supplementary Table 2 List of differentially expressed genes across injury time points. Differential expression analysis was performed paired-wise at the level of single cell types for each time point against the uninjured sample. The comparison is reported in the ‘comparison’ column. Each sheet corresponds to a different cell type. The table reports *P* values (p_val) and *P* values adjusted for multiple testing (p_val_adj). Positive values in the average log-transformed FC (avg_logFC) correspond to genes enriched in the injured condition, whereas negative values belong to genes enriched in the uninjured sample.

Supplementary Table 3 List of the top GO terms enriched for each module of genes dynamically expressed after injury. Genes in each module were clustered based on their activation pattern (that is, enriched in uninjured, acute or chronic phases).

Supplementary Table 4 List of patterns and transcription factor binding sites identified by our cell-type- and injury-specific models and recognized by MoDiSCo. The table reports the number of seqlets found in each set of peaks analyzed (num_seqlets), as well as the total number of peaks (total_peaks). It also reports matches of the motifs to known transcription factors (match, qval), along with information about the transcription factor expression in the population under investigation (tf_expressed).

Supplementary Table 5 List of IREN5 mutant sequences tested in Fig. 7.

## Data Availability

All sequencing data generated are publicly available in the Gene Expression Omnibus database under accession codes GSE304349, GSE304196 and GSE304399.

## Code availability

Custom code used for the analysis is available at https://github.com/LlorensLab/zamboni_et_al.
