# Supplementary material for: The regulatory code of injury-responsive enhancers enables precision cell-state targeting in the CNS
Source: Nat Neurosci. 2025 Dec 2;29(2):337–49. doi: 10.1038/s41593-025-02131-w (PMC12880913; doi:10.1038/s41593-025-02131-w)
Supplement: Supplementary file 1 — Supplementary Figs. 1–5. [file 41593_2025_2131_MOESM1_ESM.pdf]

nature neuroscience

Supplementary information https://doi.org/10.1038/s41593-025-02131-w

The regulatory code of injury-responsive enhancers enables precision cell-state targeting in the CNS

In the format provided by the authors and unedited

Supplementary Information

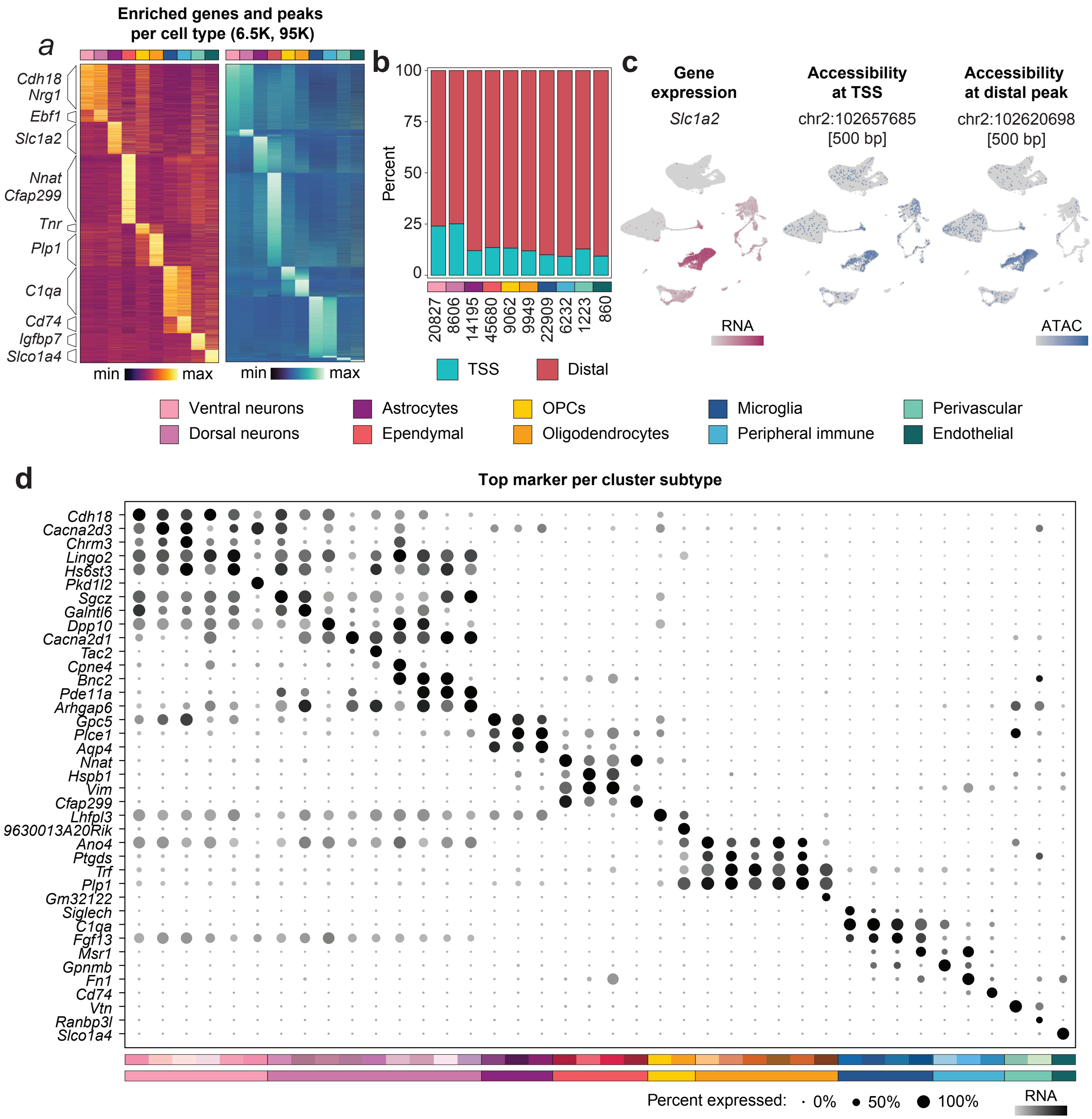
a
Enriched genes and peaks per cell type (6.5K, 95K)
Cdh18
Nrg1
Ebf1
Slc1a2
Nnat
Cfap299
Tnr
Plp1
C1qa
Cd74
Igfbp7
Slco1a4
min
max
min
max
b
Percent
100
75
50
25
0
20827
8606
14195
45680
9062
9949
22909
6232
1223
860
TSS
Distal
c
Gene expression
Slc1a2
Accessibility at TSS
chr2:102657685 [500 bp]
Accessibility at distal peak
chr2:102620698 [500 bp]
RNA
ATAC
Ventral neurons
Dorsal neurons
Astrocytes
Ependymal
OPCs
Oligodendrocytes
Microglia
Peripheral immune
Perivascular
Endothelial
d
Top marker per cluster subtype
Cdh18
Cacna2d3
Chrm3
Lingo2
Hs6st3
Pkd1l2
Sgcz
Galntl6
Dpp10
Cacna2d1
Tac2
Cpne4
Bnc2
Pde11a
Arhgap6
Gpc5
Plce1
Aqp4
Nnat
Hspb1
Vim
Cfap299
Lhfpl3
9630013A20Rik
Ano4
Ptgds
Trf
Plp1
Gm32122
Siglech
C1qa
Fgf13
Msr1
Gpnmb
Fn1
Cd74
Vtn
Ranbp3l
Slco1a4
Percent expressed:
0%
50%
100%
RNA

Supplementary Fig. 1. Cell type and subtype markers.

a, Heatmap displaying average scaled expression (left) and chromatin accessibility (right) for cell type markers from the complete dataset.

b, Barplot reporting the proportion of differentially accessible peaks mapping to either transcription start sites (TSS) or to distal genomic regions, for each cell type. Numbers reported below reflect the total number of peaks detected.

c, Gene expression, chromatin accessibility at the TSS, and at a distal enhancer for Slc1a2.

d, Dotplot displaying the average gene expression level for top markers of cell subtypes. Colored bars reflect cell subtypes (above) and cell types (below) and are matching the clusters computed on the multiomic data in Figure **1b**. Dots are colored based on the gene expression level and dot size represents the percentage of cells expressing the gene.

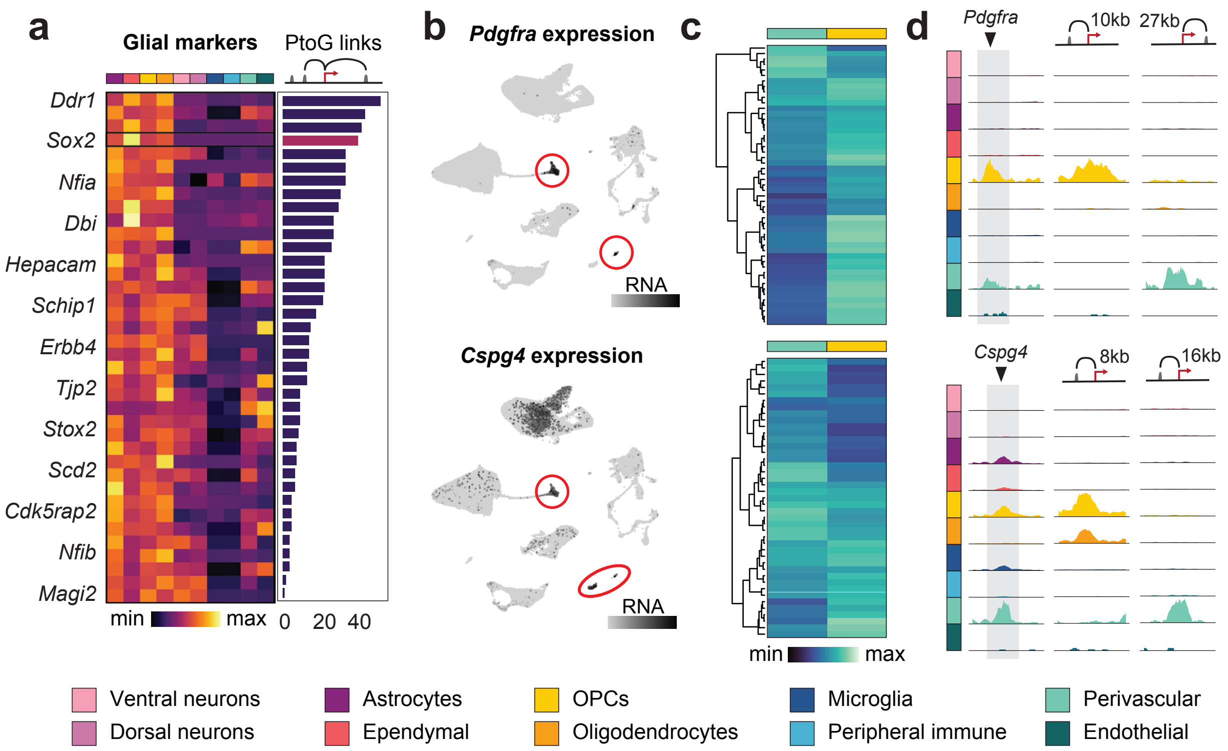

a
Glial markers
PtoG links
Ddr1
Sox2
Nfia
Dbi
Hepacam
Schip1
Erbb4
Tjp2
Stox2
Scd2
Cdk5rap2
Nfib
Magi2
min
max
0
20
40
b
Pdgfra expression
RNA
Cspg4 expression
RNA
c
min
max
d
Pdgfra
10kb
27kb
Cspg4
8kb
16kb
Ventral neurons
Dorsal neurons
Astrocytes
Ependymal
OPCs
Oligodendrocytes
Microglia
Peripheral immune
Perivascular
Endothelial

Supplementary Fig. 2. Shared gene expression is linked to cell type-specific regulatory elements.
a, On the left, the heatmap displays the average scaled gene expression of markers shared across all glial cell types. On the right, the barplot reports the number of peaks linked to each marker (peak-to-gene links, PtoG). Sox2 gene expression profile and number of linked peaks are highlighted and linked to Figure **1i-k**.
b, Feature plot displaying the gene expression levels of Pdgfra (above) and Cspg4 (below).
c, Heatmaps presenting the average scaled chromatin accessibility of peaks linked to Pdgfra (above) and Cspg4 (below) for oligodendrocyte progenitors and perivascular cells.
d, Coverage tracks of the transcription start site (TSS) and two linked distal regions for Pdgfra (above) and Cspg4 (below). Tracks are split by cell type.

a

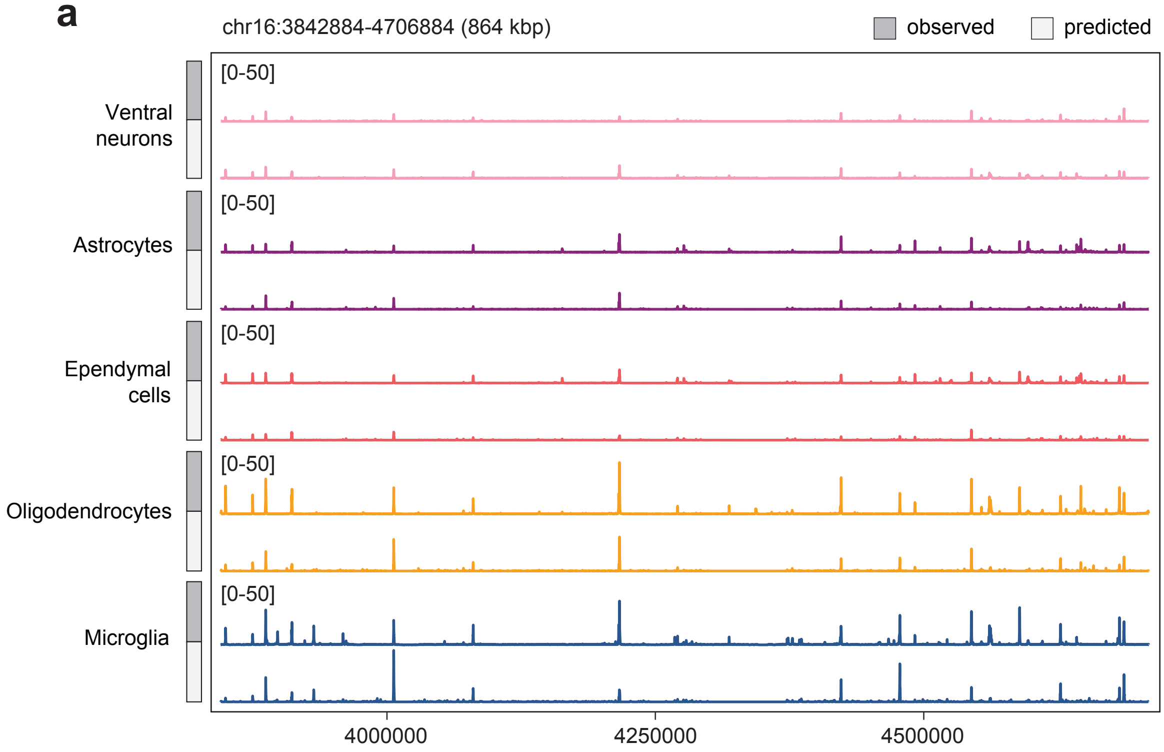

chr16:3842884-4706884 (864 kbp)
observed
predicted
Ventral neurons
Astrocytes
Ependymal cells
Oligodendrocytes
Microglia
[0-50]
[0-50]
[0-50]
[0-50]
[0-50]
4000000
4250000
4500000

Supplementary Fig. 3. Observed and predicted chromatin accessibility profiles.

a, Coverage tracks of observed and predicted chromatin accessibility over a large genomic region (864 kbp). Tracks are split by cell type and based on whether they are reporting observed or predicted profiles. In brackets are reported the y-axis limits corresponding to the read counts.

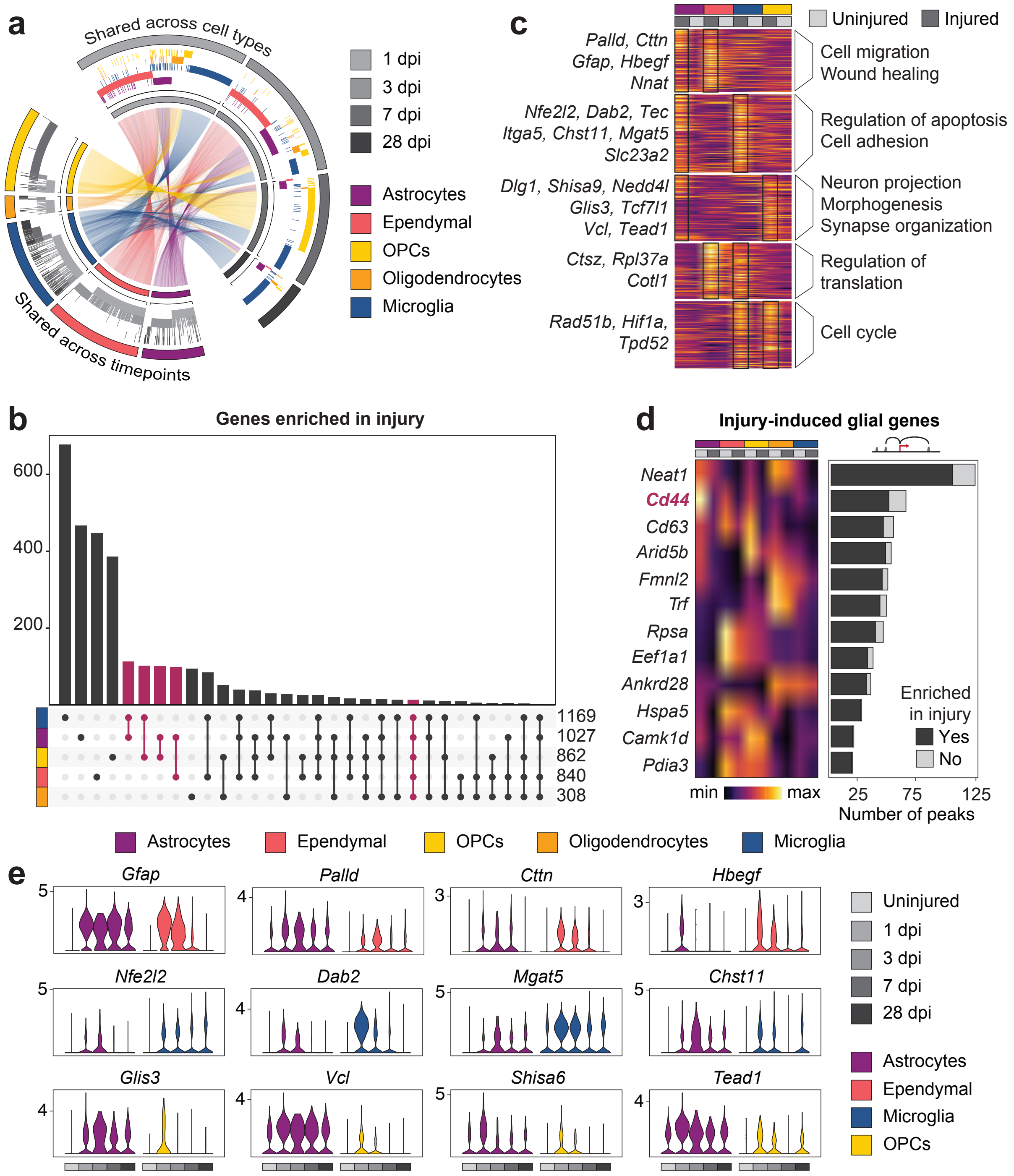
a
Shared across cell types
Shared across timepoints
1 dpi
3 dpi
7 dpi
28 dpi
Astrocytes
Ependymal
OPCs
Oligodendrocytes
Microglia
c
Uninjured
Injured
Palld, Cttn
Gfap, Hbegf
Nnat
Nfe2l2, Dab2, Tec
Itga5, Chst11, Mgat5
Slc23a2
Dlg1, Shisa9, Nedd4l
Glis3, Tcf7l1
Vcl, Tead1
Ctsz, Rpl37a
Cotl1
Rad51b, Hif1a,
Tpd52
Cell migration
Wound healing
Regulation of apoptosis
Cell adhesion
Neuron projection
Morphogenesis
Synapse organization
Regulation of
translation
Cell cycle
b
Genes enriched in injury
600
400
200
1169
1027
862
840
308
d
Injury-induced glial genes
Neat1
Cd44
Cd63
Arid5b
Fmnl2
Trf
Rpsa
Eef1a1
Ankrd28
Hspa5
Camk1d
Pdia3
min
max
25
75
125
Number of peaks
Enriched
in injury
Yes
No
Astrocytes
Ependymal
OPCs
Oligodendrocytes
Microglia
e
Gfap
Palld
Cttn
Hbegf
Nfe2l2
Dab2
Mgat5
Chst11
Glis3
Vcl
Shisa6
Tead1
Uninjured
1 dpi
3 dpi
7 dpi
28 dpi
Astrocytes
Ependymal
Microglia
OPCs

Supplementary Fig. 4. Shared injury-induced gene expression programs across glial cells.
a, Circos plots representing the sharedness of injury-enriched genes across timepoints and cell types.
b, Upset plot displaying the number of genes upregulated after injury, which are unique or shared across glial cell types. In purple, genes are highlighted which are enriched in pairs of cell types or shared across all glial cells. Numbers at the bottom reflect the total number of enriched genes per cluster.
c, Heatmap of injury-enriched genes shared between pairs of cell types. Genes are grouped according to cell type (colored bar above) and condition (i.e., uninjured and injured, colored bar below) and the pairs are highlighted by the black boxes. On the left, examples of genes belonging to each group of features are displayed, and on the right, examples of enriched gene ontology terms are highlighted.
d, Heatmap showing the average scaled expression of injury-induced genes shared across all glial cells. Genes are plotted for each cell type (colored bar above) and injury condition (colored bar below). On the right, barplots report the total number of peaks linked to the genes in the heatmap, which are colored based on whether they are themselves enriched after injury.
e, Violin plots displaying gene expression profiles of representative genes showing pairwise enrichment after injury.

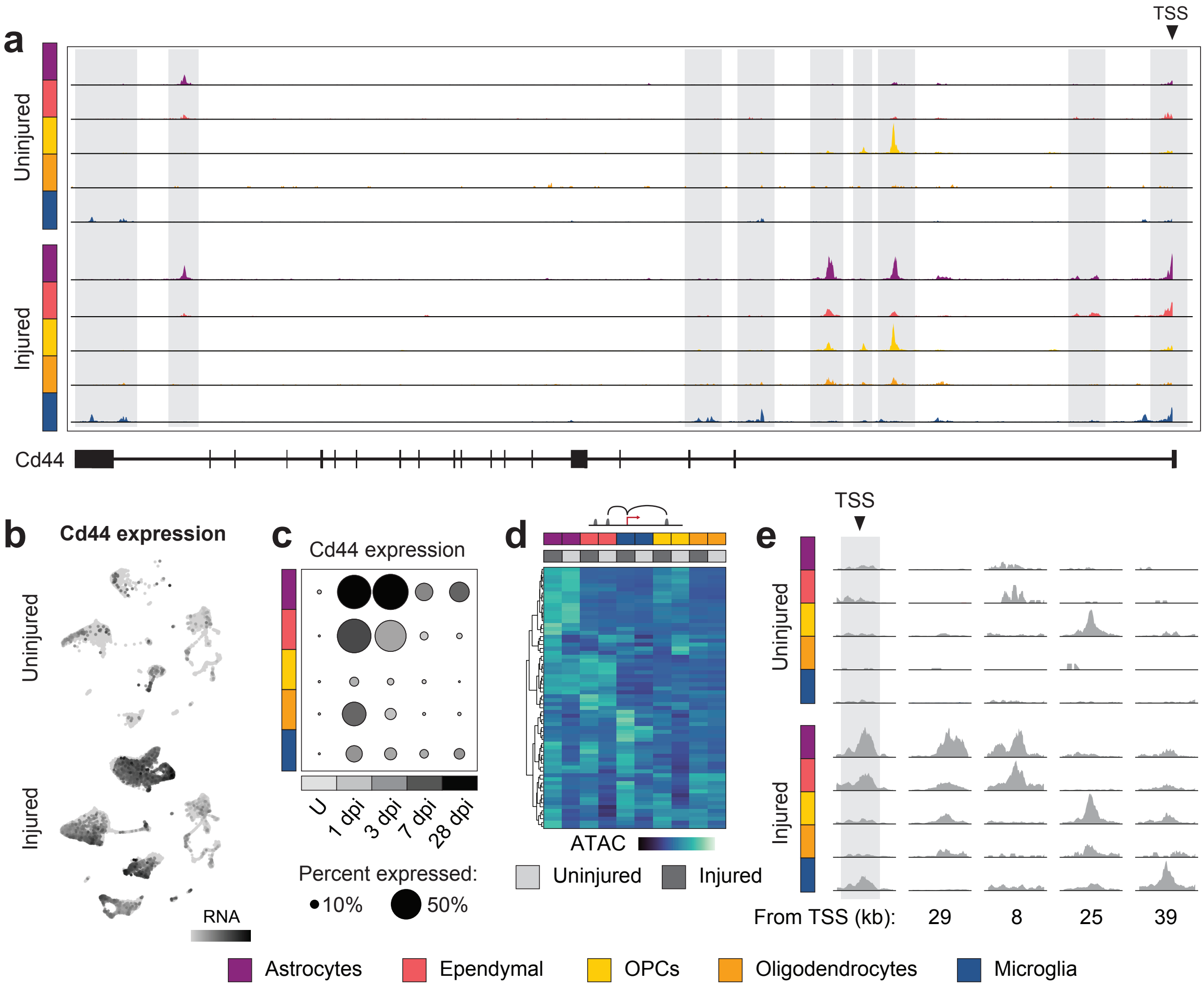

a
TSS
Uninjured
Injured
Cd44
b
Cd44 expression
Uninjured
Injured
RNA
c
Cd44 expression
U
1 dpi
3 dpi
7 dpi
28 dpi
Percent expressed:
10%
50%
d
ATAC
Uninjured
Injured
e
TSS
Uninjured
Injured
From TSS (kb):
29
8
25
39
Astrocytes
Ependymal
OPCs
Oligodendrocytes
Microglia

Supplementary Fig. 5. Injury-dependent regulation of Cd44 across glial cells.

a, Coverage track of the Cd44 gene body. Accessibility of loci is represented cluster-wise and split by injury condition. Transcriptional start site (TSS) and distal regulatory elements are highlighted (grey areas).

b, Feature plot displaying gene expression of Cd44 in uninjured and injured samples.

c, Dotplot reporting the average Cd44 expression level for each glial cell type and timepoint after spinal cord injury. Dots are colored based on the average score (white to black) and percentage of cells expressing the gene is represented by the relative dot size.

d, Heatmap representing average scaled accessibility across glia cells and conditions (i.e., uninjured and injured) of peaks linked to Cd44.

e, Coverage track of the Cd44 transcription start site (TSS) along with representative examples of distal peaks linked to Cd44 and enriched in different cell types and states. Distance from the TSS of the distal genomic regions is reported (in kb) below the plots.
