# Supplementary material for: The regulatory code of injury-responsive enhancers enables precision cell-state targeting in the CNS
Source: Nat Neurosci. 2025 Dec 2;29(2):337–49. doi: 10.1038/s41593-025-02131-w (PMC12880913; doi:10.1038/s41593-025-02131-w)
Supplement: Supplementary file 2 — Reporting Summary [file 41593_2025_2131_MOESM2_ESM.pdf]

nature portfolio

Corresponding author(s): Enric Llorens-Bobadilla (enric.llorens@ki.se)

Last updated by author(s): Sep 19, 2025

Reporting Summary

Nature Portfolio wishes to improve the reproducibility of the work that we publish. This form provides structure for consistency and transparency in reporting. For further information on Nature Portfolio policies, see our Editorial Policies and the Editorial Policy Checklist.

Statistics

For all statistical analyses, confirm that the following items are present in the figure legend, table legend, main text, or Methods section.

n/a	Confirmed	
☐	☒	The exact sample size (*n*) for each experimental group/condition, given as a discrete number and unit of measurement
☐	☒	A statement on whether measurements were taken from distinct samples or whether the same sample was measured repeatedly
☐	☒	The statistical test(s) used AND whether they are one- or two-sided<br>*Only common tests should be described solely by name; describe more complex techniques in the Methods section.*
☐	☒	A description of all covariates tested
☐	☒	A description of any assumptions or corrections, such as tests of normality and adjustment for multiple comparisons
☐	☒	A full description of the statistical parameters including central tendency (e.g. means) or other basic estimates (e.g. regression coefficient) AND variation (e.g. standard deviation) or associated estimates of uncertainty (e.g. confidence intervals)
☐	☒	For null hypothesis testing, the test statistic (e.g. *F*, *t*, *r*) with confidence intervals, effect sizes, degrees of freedom and *P* value noted<br>*Give P values as exact values whenever suitable.*
☐	☒	For Bayesian analysis, information on the choice of priors and Markov chain Monte Carlo settings
☐	☒	For hierarchical and complex designs, identification of the appropriate level for tests and full reporting of outcomes
☐	☒	Estimates of effect sizes (e.g. Cohen's *d*, Pearson's *r*), indicating how they were calculated

Our web collection on statistics for biologists contains articles on many of the points above.

Software and code

Policy information about availability of computer code

Data collection	Data was collected with Illumina NextSeq and MGI sequencers and softwares.<br>Imaging data were acquired on a LSM700 ZEISS confocal microscope and processed on the ZEISS ZEN software.
Data analysis	Data analysis was performed with a combination of published packages and custom scripts, all of which can be found at https://github.com/LlorensLab/zamboni_et_al<br>The softwares used here are:<br>CellRanger-ARC (2.0.2)<br>CellRanger (8.0.1)<br>MACS2 (2.2.7.1)<br>R (4.4.1)<br>Fiji (2.14.0)<br>deML (1.1.4)<br>CellBender (0.3.2)<br>ChromBPNet (0.1.7)<br>Tensorflow (2.8.0)<br>Keras (2.8.0)<br>bedtools (2.30.0)<br>DeepLIFT (0.6.13)<br>TF-MoDISco (2.0.7)<br>SCENIC+ (1.0a1)<br>pycisTopic (2.0a0)

April 2023

Tangermeme (0.4.0)

R packages used are:
Seurat (4.3.0)
Signac (1.12.0)
ArchR (1.0.2)
EnsDb.Mmusculus.v79 (2.99.0)
DoubletFinder (2.0.3)
scDblFinder (1.16.0)
TCseq (1.28.0)
gprofiler2 (0.2.3)
ggplot2 (3.4.4)
pheatmap (1.0.12)
circlize (0.4.16)

For manuscripts utilizing custom algorithms or software that are central to the research but not yet described in published literature, software must be made available to editors and reviewers. We strongly encourage code deposition in a community repository (e.g. GitHub). See the Nature Portfolio guidelines for submitting code & software for further information.

Data

Policy information about availability of data

All manuscripts must include a data availability statement. This statement should provide the following information, where applicable:

- Accession codes, unique identifiers, or web links for publicly available datasets
- A description of any restrictions on data availability
- For clinical datasets or third party data, please ensure that the statement adheres to our policy

Raw data and processed count matrices can be found using the GEO accession codes GSE304349, GSE304196, and GSE304399

Reference genomes used to map the mouse data were obtained by 10x Genomics as part of the standard Cellranger-ARC and Cellranger pipelines (v2.0.2)

Research involving human participants, their data, or biological material

Policy information about studies with human participants or human data. See also policy information about sex, gender (identity/presentation), and sexual orientation and race, ethnicity and racism.

Reporting on sex and gender	*Use the terms sex (biological attribute) and gender (shaped by social and cultural circumstances) carefully in order to avoid confusing both terms. Indicate if findings apply to only one sex or gender; describe whether sex and gender were considered in study design; whether sex and/or gender was determined based on self-reporting or assigned and methods used.*<br>*Provide in the source data disaggregated sex and gender data, where this information has been collected, and if consent has been obtained for sharing of individual-level data; provide overall numbers in this Reporting Summary. Please state if this information has not been collected.*<br>*Report sex- and gender-based analyses where performed, justify reasons for lack of sex- and gender-based analysis.*
Reporting on race, ethnicity, or other socially relevant groupings	*Please specify the socially constructed or socially relevant categorization variable(s) used in your manuscript and explain why they were used. Please note that such variables should not be used as proxies for other socially constructed/relevant variables (for example, race or ethnicity should not be used as a proxy for socioeconomic status).*<br>*Provide clear definitions of the relevant terms used, how they were provided (by the participants/respondents, the researchers, or third parties), and the method(s) used to classify people into the different categories (e.g. self-report, census or administrative data, social media data, etc.)*<br>*Please provide details about how you controlled for confounding variables in your analyses.*
Population characteristics	*Describe the covariate-relevant population characteristics of the human research participants (e.g. age, genotypic information, past and current diagnosis and treatment categories). If you filled out the behavioural & social sciences study design questions and have nothing to add here, write "See above."*
Recruitment	*Describe how participants were recruited. Outline any potential self-selection bias or other biases that may be present and how these are likely to impact results.*
Ethics oversight	*Identify the organization(s) that approved the study protocol.*

Note that full information on the approval of the study protocol must also be provided in the manuscript.

Field-specific reporting

Please select the one below that is the best fit for your research. If you are not sure, read the appropriate sections before making your selection.

☒ Life sciences ☐ Behavioural & social sciences ☐ Ecological, evolutionary & environmental sciences

For a reference copy of the document with all sections, see nature.com/documents/nr-reporting-summary-flat.pdf

Life sciences study design

All studies must disclose on these points even when the disclosure is negative.

Sample size	No sample size calculations were performed for this study, but the appropriate number of animals to allocate for each experimental condition was estimated on the basis of previous studies using the same technologies. For the sequencing experiments, 2-4 animals were pooled for each condition. More animals (n=4) were used in the samples for which we performed cell enrichment to ensure collection of appropriate number of cells . Each condition included equal numbers of female and male mice.<br>For the in vivo validation, 3-4 animals per condition were used. We used both females and males.
Data exclusions	During the surgical procedures, animals were excluded from the experiment in case of failure to meet the a priori criteria of contusion severity or post operative complications as per ethical regulations (e.g., >15% weight loss).<br>For the sequencing experiments, low quality nuclei were removed on the basis of their total counts or the proportion of mitochondrial reads.
Replication	Data were processed using different computational tools, for instance to identify peaks in the chromatin accessibility data (i.e., MACS2, Signac, ArchR). Results were compared and confirmed to have good overlap.
Randomization	Animals were randomly allocated to each experimental condition
Blinding	Whenever possible, quantifications were performed blinded to experimental condition.<br>For the sequencing analysis, tools allowing unsupervised clustering were used (e.g., Seurat).

Reporting for specific materials, systems and methods

We require information from authors about some types of materials, experimental systems and methods used in many studies. Here, indicate whether each material, system or method listed is relevant to your study. If you are not sure if a list item applies to your research, read the appropriate section before selecting a response.

Materials & experimental systems

n/a	Involved in the study	
☐	☒	Antibodies
☒	☐	Eukaryotic cell lines
☒	☐	Palaeontology and archaeology
☐	☒	Animals and other organisms
☒	☐	Clinical data
☒	☐	Dual use research of concern
☒	☐	Plants

Methods

n/a	Involved in the study	
☒	☐	ChIP-seq
☒	☐	Flow cytometry
☒	☐	MRI-based neuroimaging

Antibodies

Antibodies used	Primary antibodies<br>GFP (chicken, 1:1000, Aves, GFP1010)<br>RFP (rabbit, 1:1000, Rockland, 600-401-379)<br>Gfap (rabbit, 1:500, Dako, Z0334)<br>Sox9 (goat, 1:250, R&D, AF3075)<br>Sox9 (rabbit 1:100, Sigma AB5535)<br>NeuN (mouse 1:500, Millipore MAB377)<br>Sox10 (rabbit 1:200, Millipore AB5727)<br><br>Secondary antibodies<br>Donkey anti-rabbit, anti-goat, anti-chicken IgG (H+L) Highly Cross-Absorbed Secondary Antibody, Thermo<br>Guinea pig anti-rabbit secondary antibody (1:50, Novus Biologicals, NBP1-72763)
Validation	anti-GFP: 19 citations listed for immunofluorescence use in mouse tissue at https://www.citeab.com/antibodies/575207-gfp-1010-anti-green-fluorescent-protein-gfp-antibod<br>anti-RFP: multiple citations of use in mouse tissue for immunostaining on the manufacturer's website (e.g., PMC10844855)<br>anti-Gfap: 66 citations listed for immunofluorescence use on mouse tissue at https://www.citeab.com/antibodies/2452274-z0334-glial-fibrillary-acidic-protein-gfap<br>anti-Sox9: multiple citations of use in mouse tissue for immunostaining on the manufacturer's website (e.g., PMID35294885)

Animals and other research organisms

Policy information about studies involving animals; ARRIVE guidelines recommended for reporting animal research, and Sex and Gender in Research

Laboratory animals	Wild type mice from the train C57BL/6JR were obtained from Janvier and used at the age of 2 to 6 months old.<br>Transgenic mouse lines used includer Foxj1-tdTomato and Cx30-tdTomato reporter mice.<br>Mice were housed with a standard light/dark cycle and availability of food and water ad libitum.
Wild animals	The study did not involve wild animals
Reporting on sex	For each experimental condition, samples were composed by both female and male mice in equal numbers.
Field-collected samples	The study did not involve field-collected samples
Ethics oversight	Experimental procedures were performed in compliance with governmental and ethical specifications approved by the Karolinska Institute (Stockholm, Sweden) and the Stockholms Norra Djurförsöksetiska Nämnd under the ethical permit number 20785/2020

Note that full information on the approval of the study protocol must also be provided in the manuscript.

Plants

Seed stocks	*Report on the source of all seed stocks or other plant material used. If applicable, state the seed stock centre and catalogue number. If plant specimens were collected from the field, describe the collection location, date and sampling procedures.*
Novel plant genotypes	*Describe the methods by which all novel plant genotypes were produced. This includes those generated by transgenic approaches, gene editing, chemical/radiation-based mutagenesis and hybridization. For transgenic lines, describe the transformation method, the number of independent lines analyzed and the generation upon which experiments were performed. For gene-edited lines, describe the editor used, the endogenous sequence targeted for editing, the targeting guide RNA sequence (if applicable) and how the editor was applied.*
Authentication	*Describe any authentication procedures for each seed stock used or novel genotype generated. Describe any experiments used to assess the effect of a mutation and, where applicable, how potential secondary effects (e.g. second site T-DNA insertions, mosiacism, off-target gene editing) were examined.*
